# Supplementary figures and images for: Creating artificial human genomes using generative neural networks
Source: PLoS Genet. 2021 Feb 4;17(2):e1009303. doi: 10.1371/journal.pgen.1009303 (PMC7861435; doi:10.1371/journal.pgen.1009303)

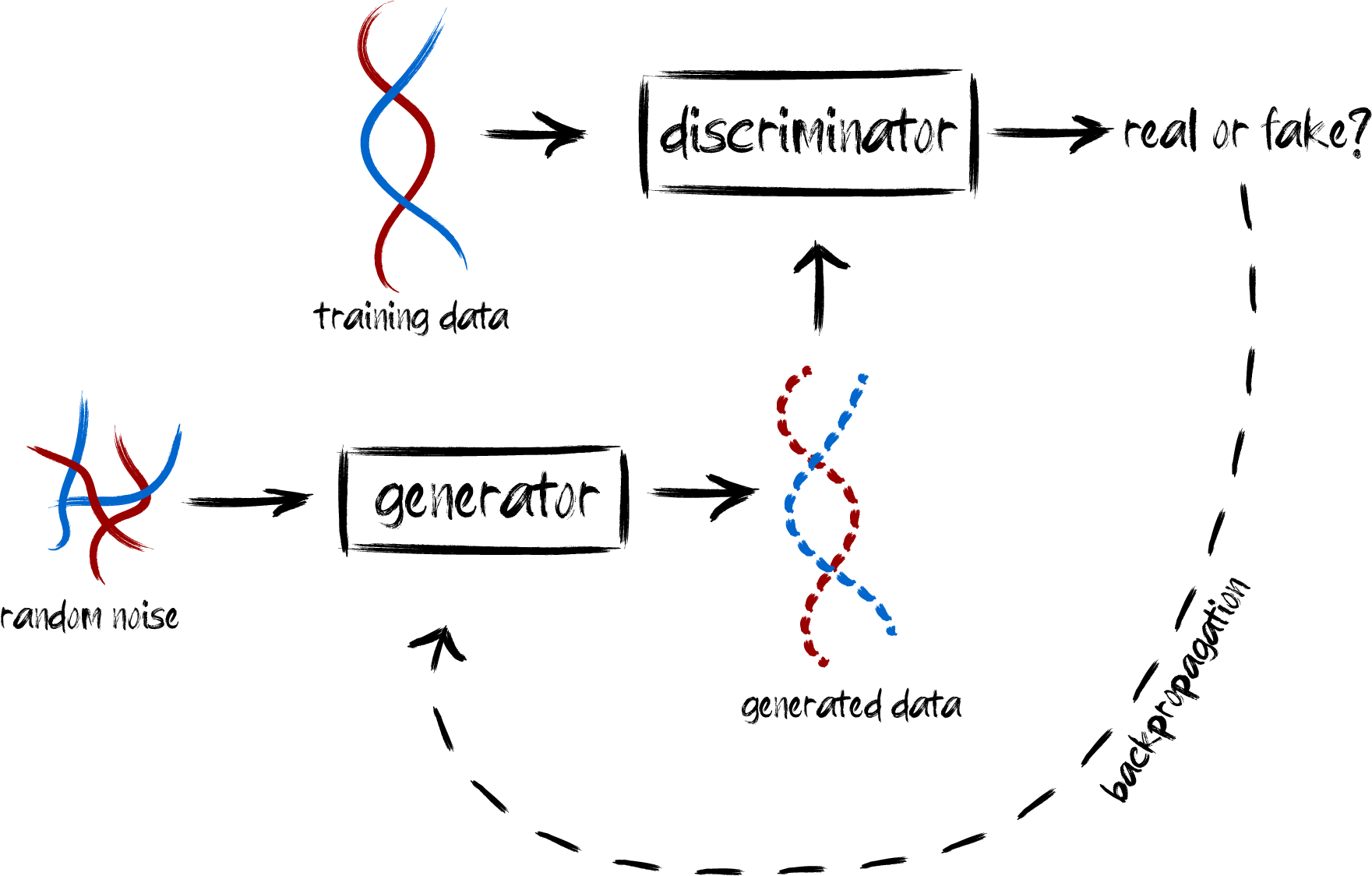

Supplement: S1 Fig — (TIF) [file pgen.1009303.s001.tif]

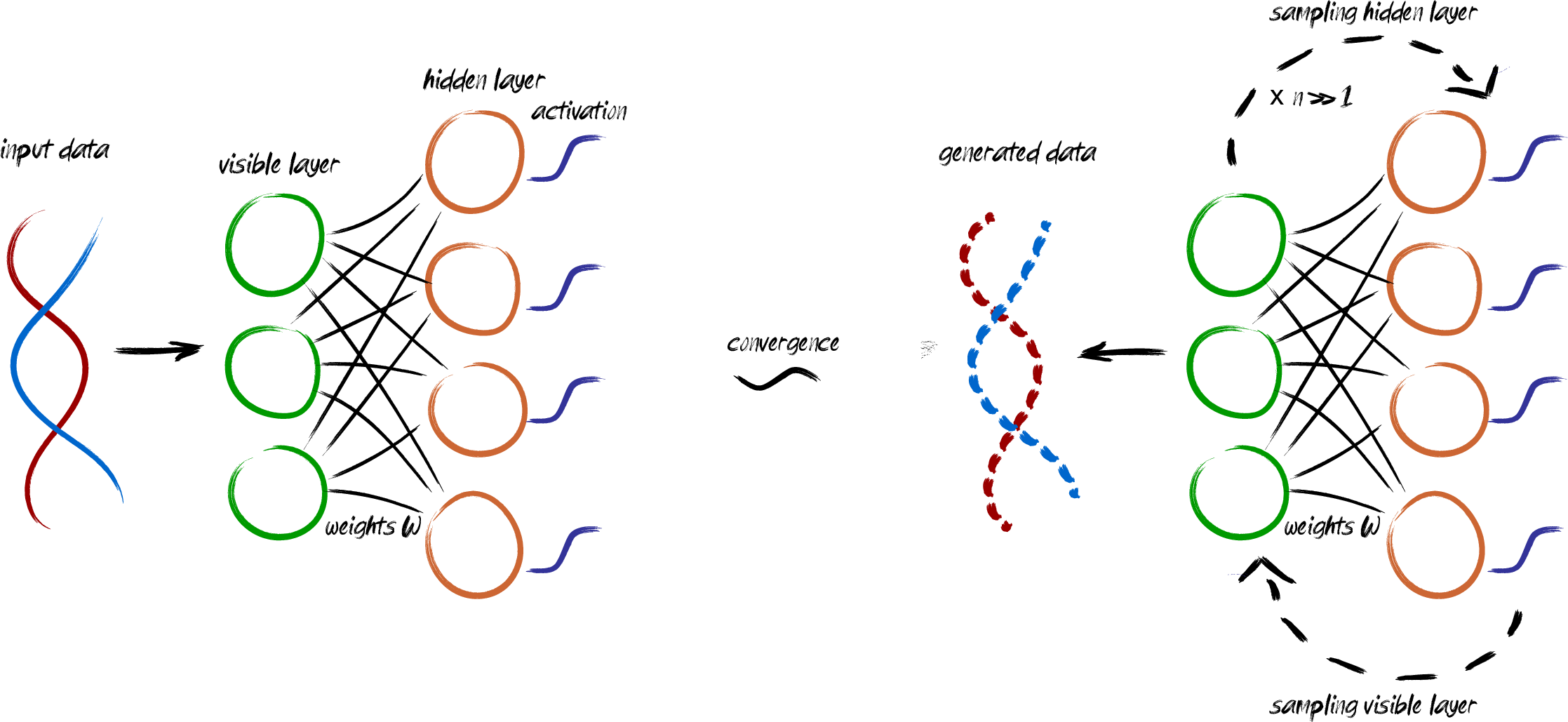

Supplement: S2 Fig — (TIF) [file pgen.1009303.s002.tif]

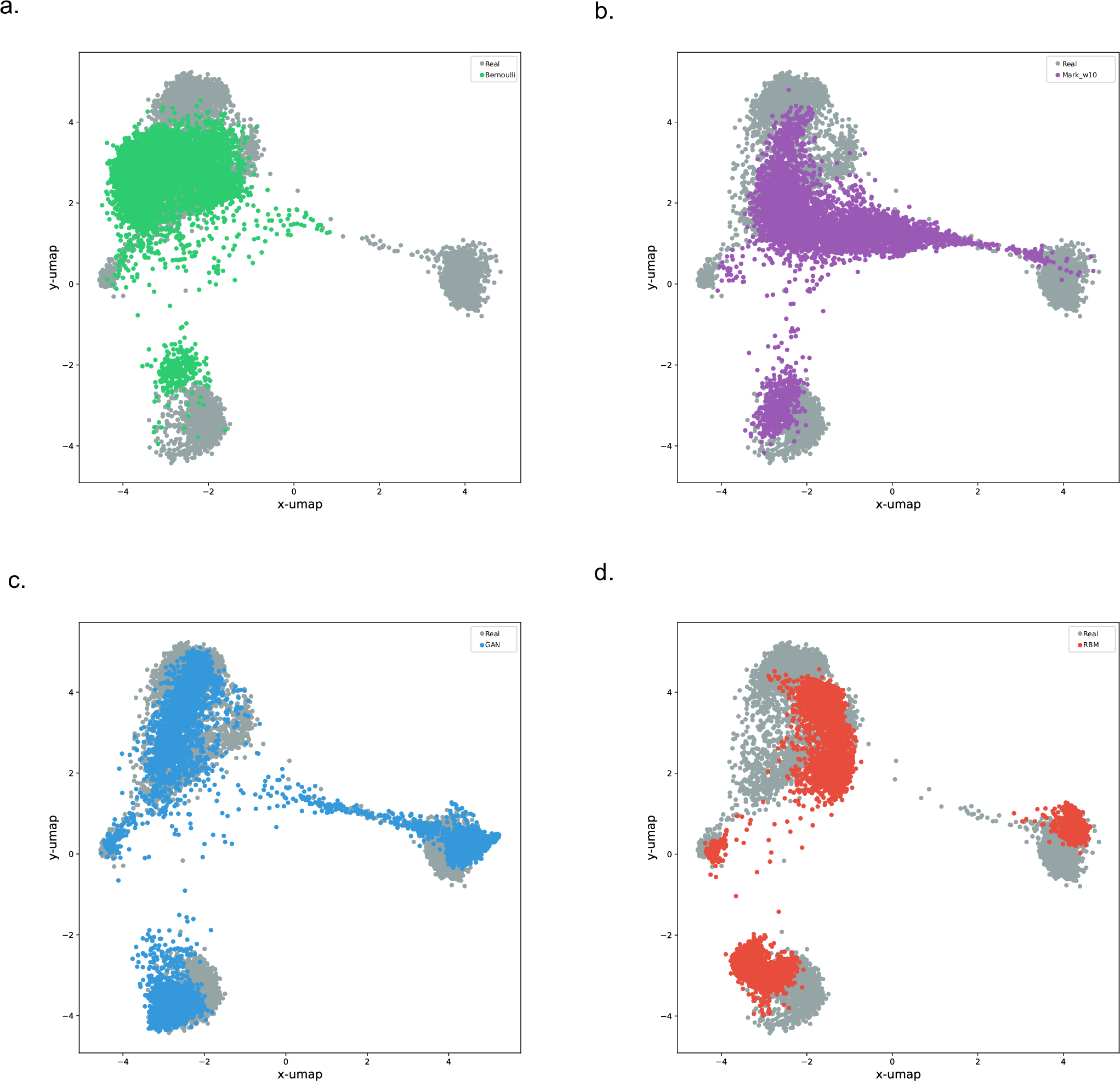

Supplement: S3 Fig — Uniform manifold approximation and projection (UMAP) of real genomes from 1000 Genomes data spanning 805 SNPs along with artificial genome counterparts created via a) Bernoulli, b) Markov chain (with 10 window length), c) GAN and d) RBM models. (TIF) [file pgen.1009303.s003.tif]

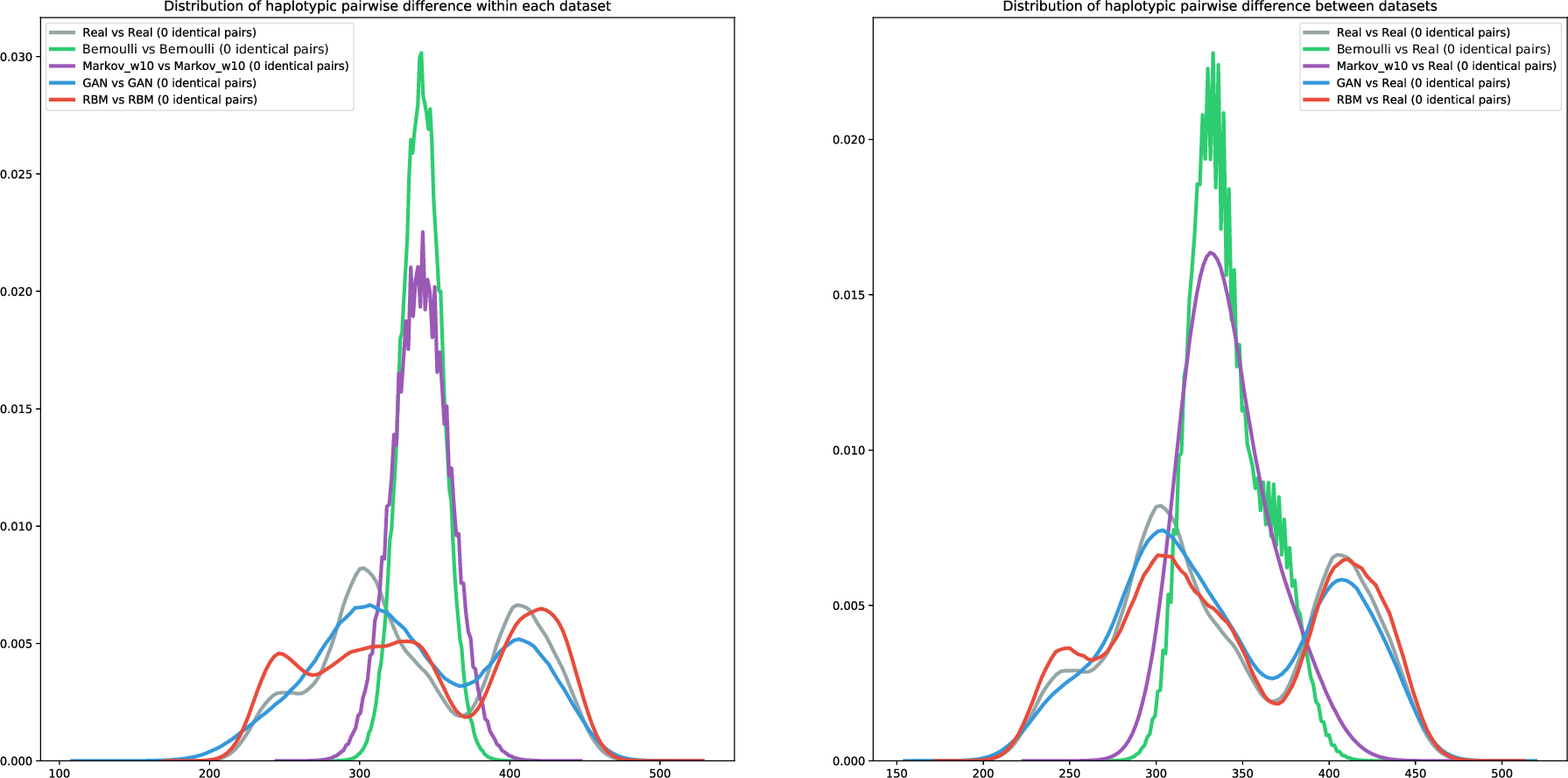

Supplement: S4 Fig — (TIF) [file pgen.1009303.s004.tif]

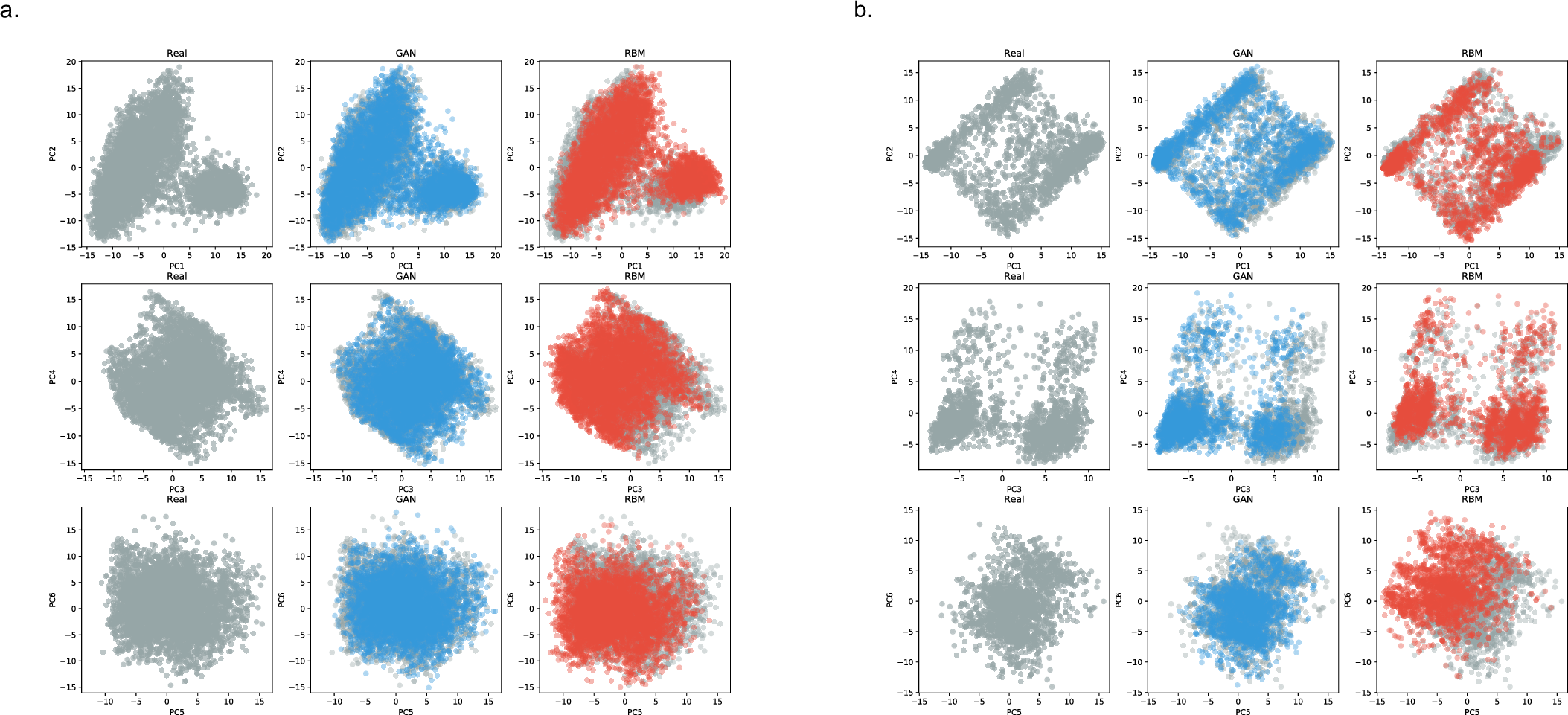

Supplement: S5 Fig — PCA of real genomes (gray) from a) 1000 Genomes data and b) Estonian Biobank spanning 10K SNPs along with artificial genome counterparts generated using GAN (blue) and RBM (red) models. (TIF) [file pgen.1009303.s005.tif]

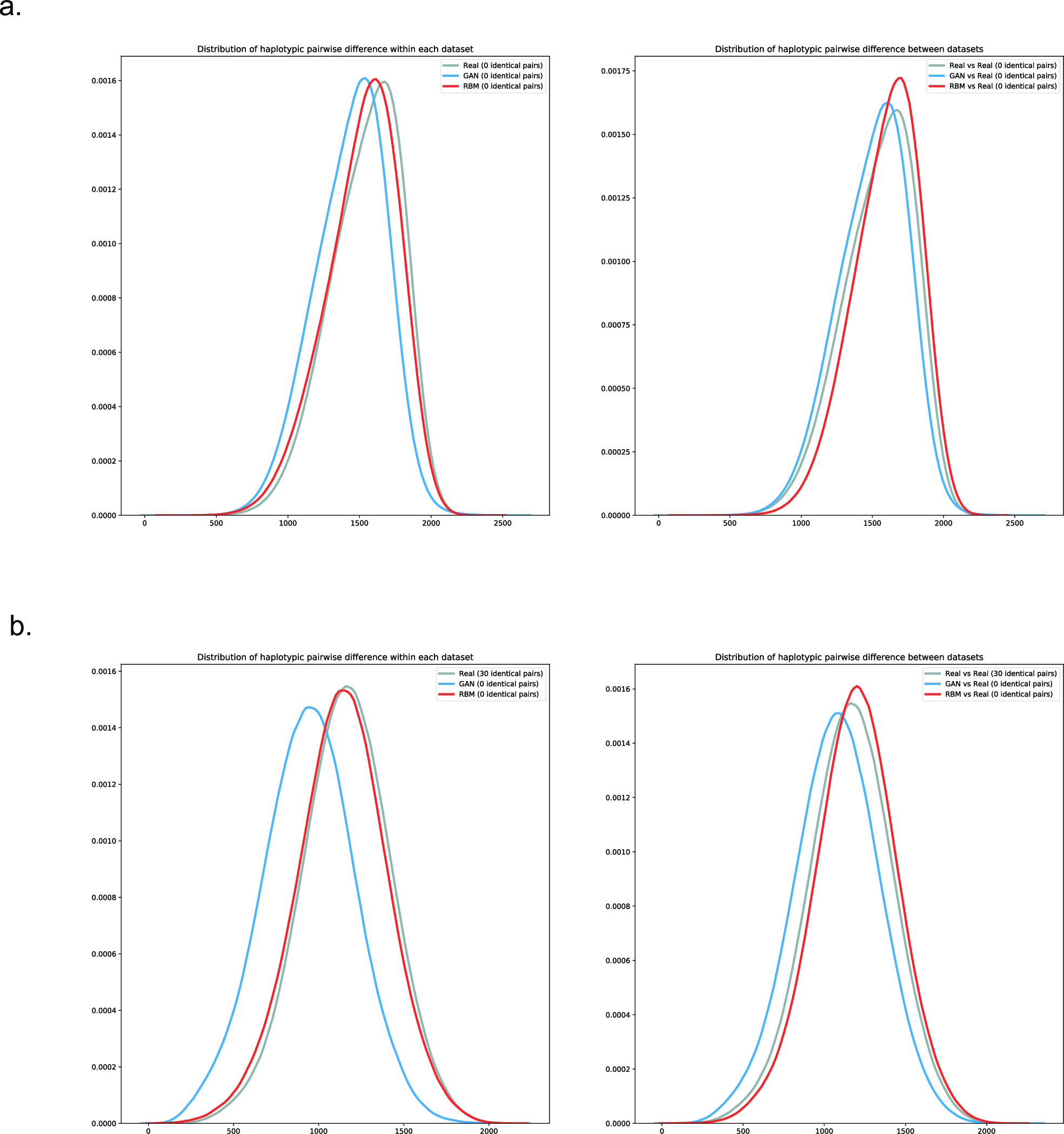

Supplement: S6 Fig — Distribution of haplotypic pairwise difference within (left) and between (right) datasets of real genomes from a) 1000 Genomes data and b) Estonian Biobank spanning 10K SNPs and artificial genome counterparts generated using GAN and RBM models. (TIF) [file pgen.1009303.s006.tif]

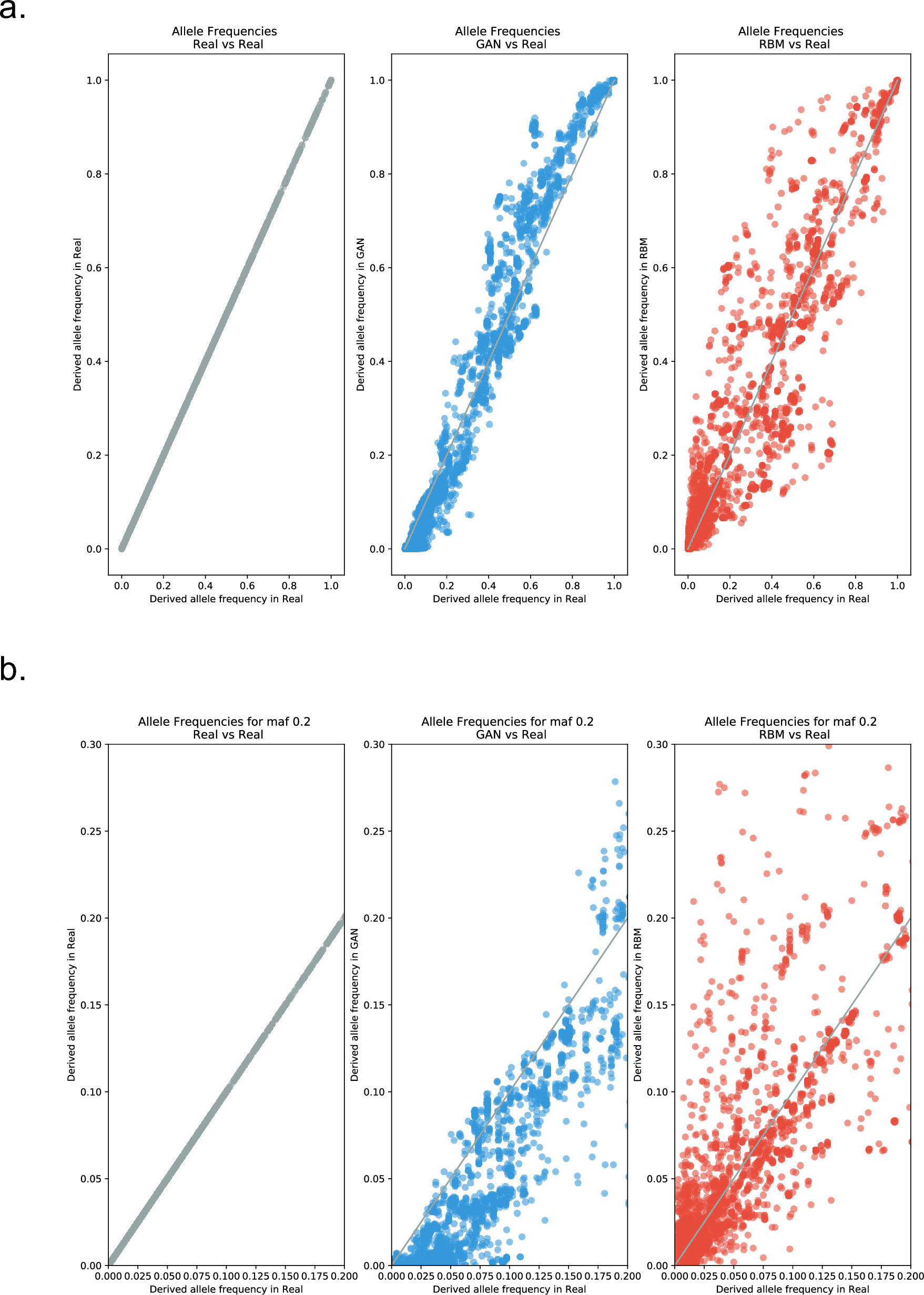

Supplement: S7 Fig — Allele frequency comparison of corresponding SNPs between real genomes from Estonian Biobank spanning 10K SNPs and artificial genome counterparts generated using GAN and RBM models as a) the whole range and b) zoomed to low frequencies. Clustering below the diagonal in the low frequency section for the GAN plot indicates insufficient representation of rare alleles in artificial genomes. (TIF) [file pgen.1009303.s007.tif]

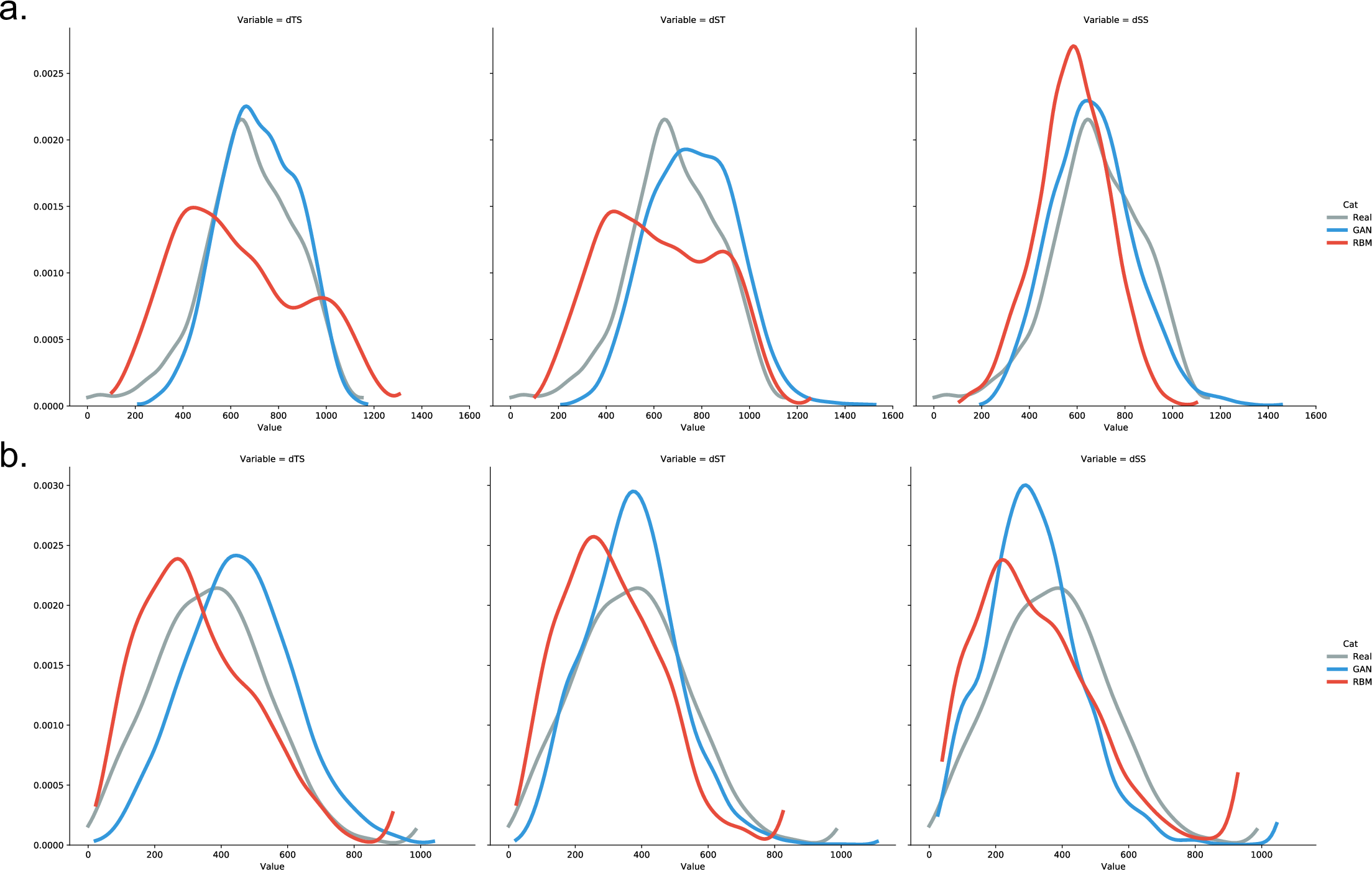

Supplement: S8 Fig — Distribution of minimum distance to the closest neighbour for real genomes from a) 1000 Genomes data and b) Estonian Biobank spanning 10K SNPs along with artificial genome counterparts generated via GAN and RBM models. (TIF) [file pgen.1009303.s008.tif]

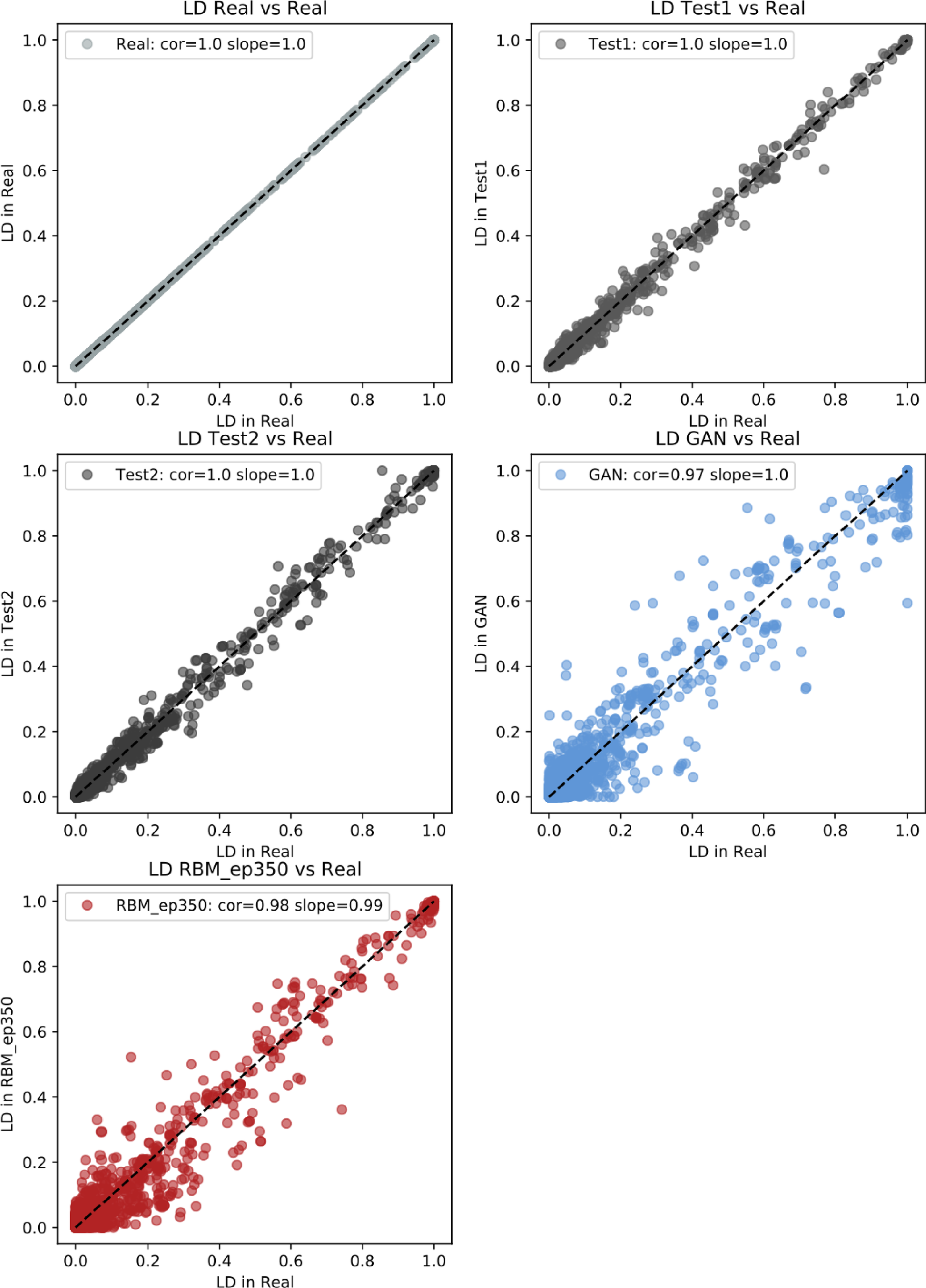

Supplement: S9 Fig — (TIF) [file pgen.1009303.s009.tif]

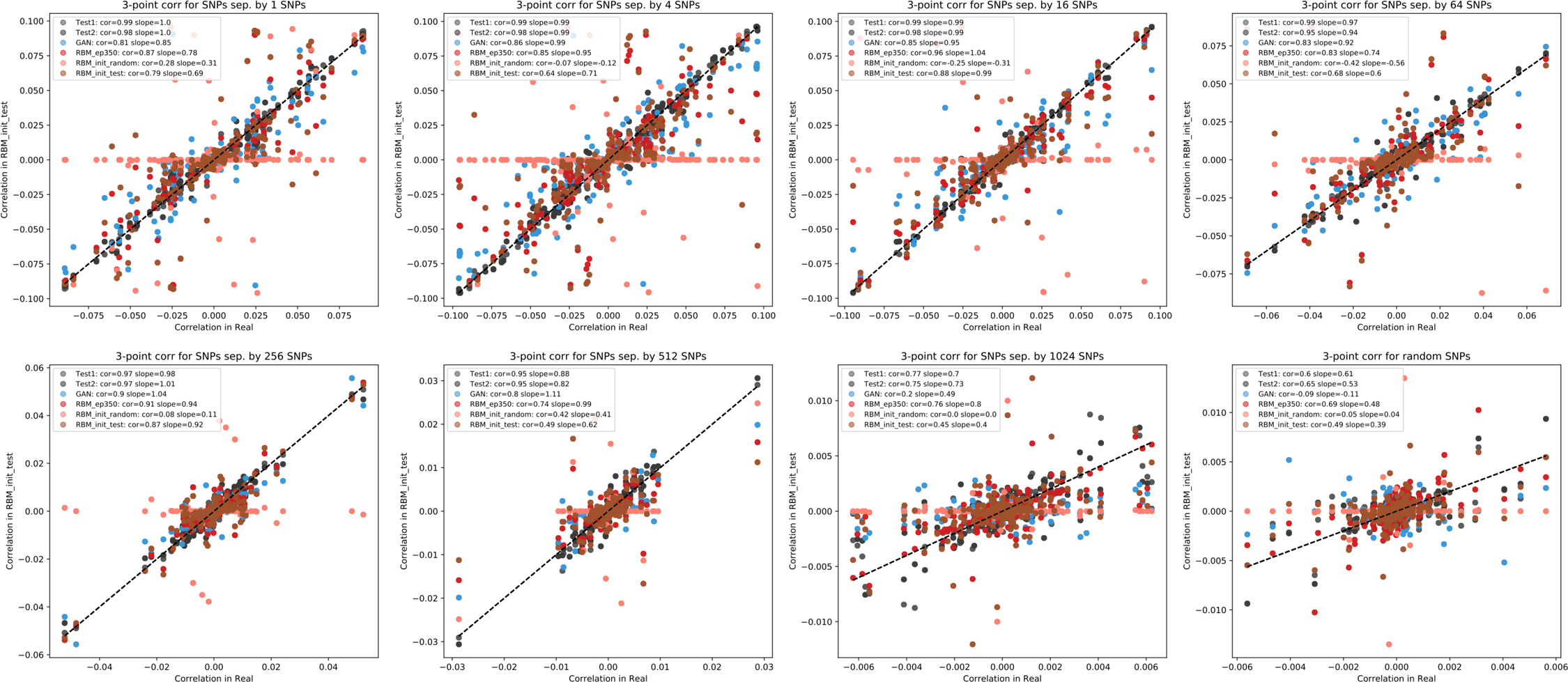

Supplement: S10 Fig — (TIF) [file pgen.1009303.s010.tif]

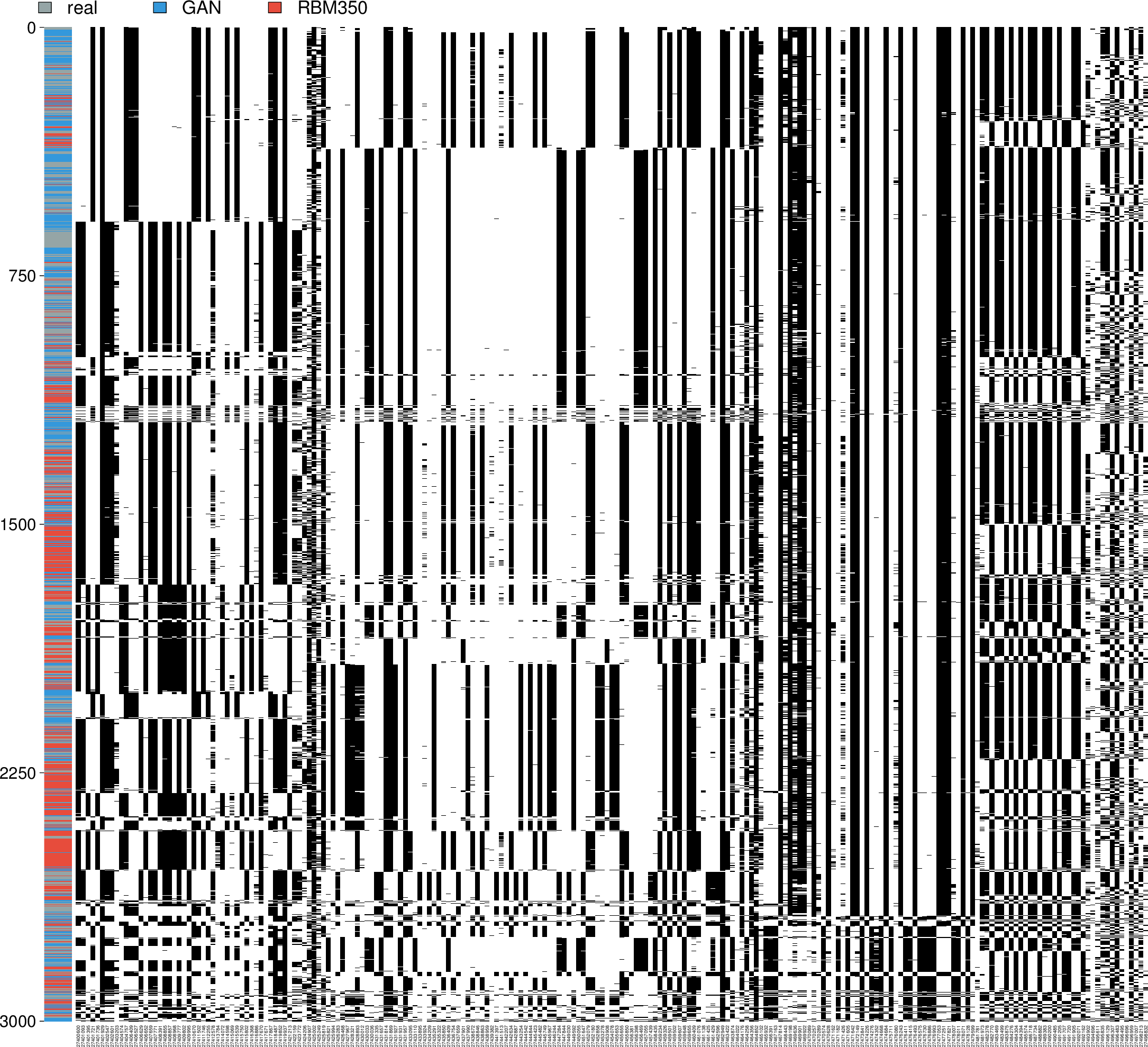

Supplement: S11 Fig — (TIF) [file pgen.1009303.s011.tif]

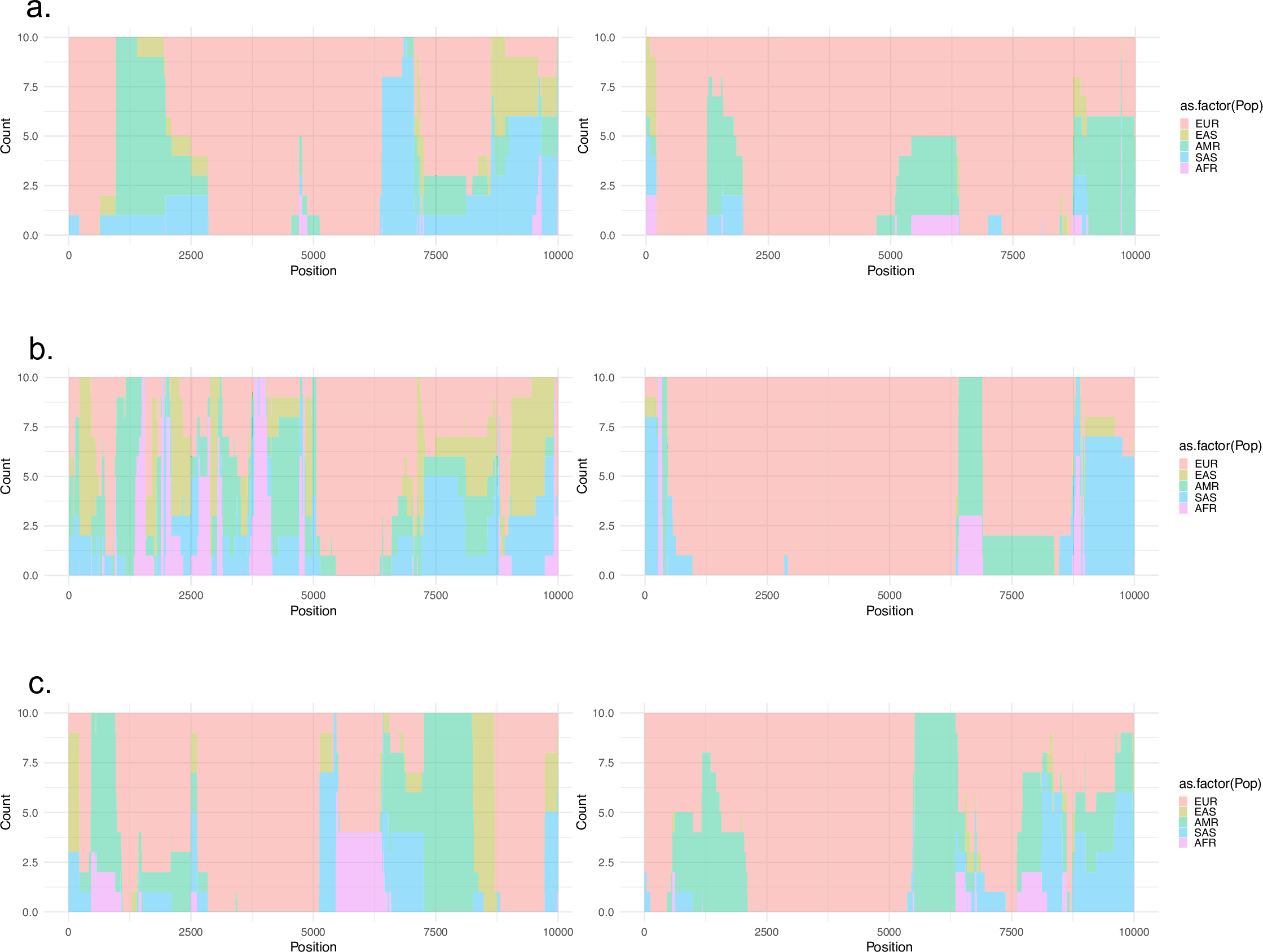

Supplement: S12 Fig — Chromosome painting of two a) real Estonian genomes, b) GAN and c) RBM artificial Estonian genomes with 1000 Genomes donors colored based on super population codes. EUR–European, EAS–East Asian, AMR–Admixed American, SAS–South Asian, AFR–African. (TIF) [file pgen.1009303.s012.tif]

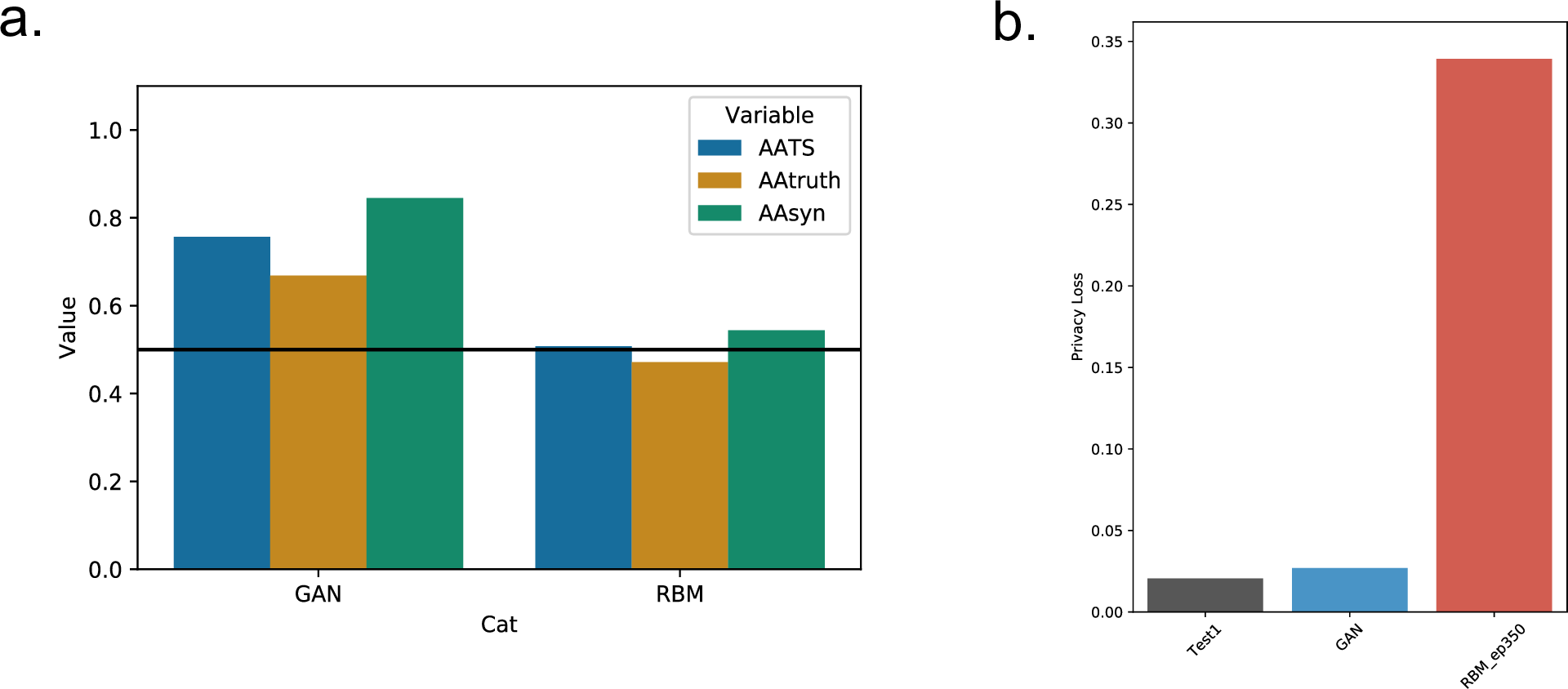

Supplement: S13 Fig — a) Nearest neighbour adversarial accuracy (AATS) scores of artificial genomes generated from Estonian Biobank. Black line indicates the optimum value whereas values below the line indicate overfitting and values above the line indicate underfitting. b) Privacy loss. Test1 is a separate set of real Estonian genomes. Positive values indicate information leakage, hence overfitting. (TIF) [file pgen.1009303.s013.tif]

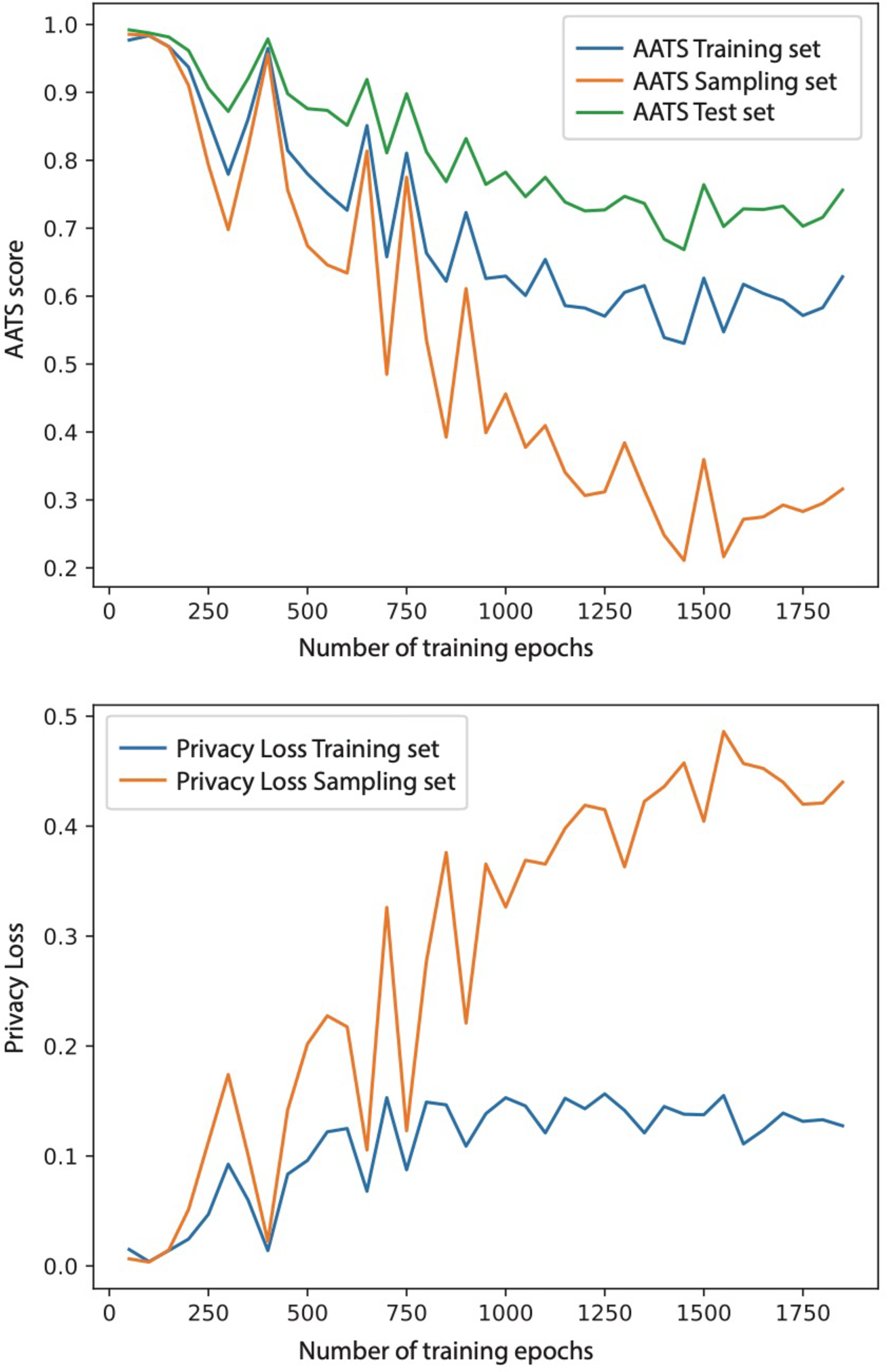

Supplement: S14 Fig — (TIF) [file pgen.1009303.s014.tif]

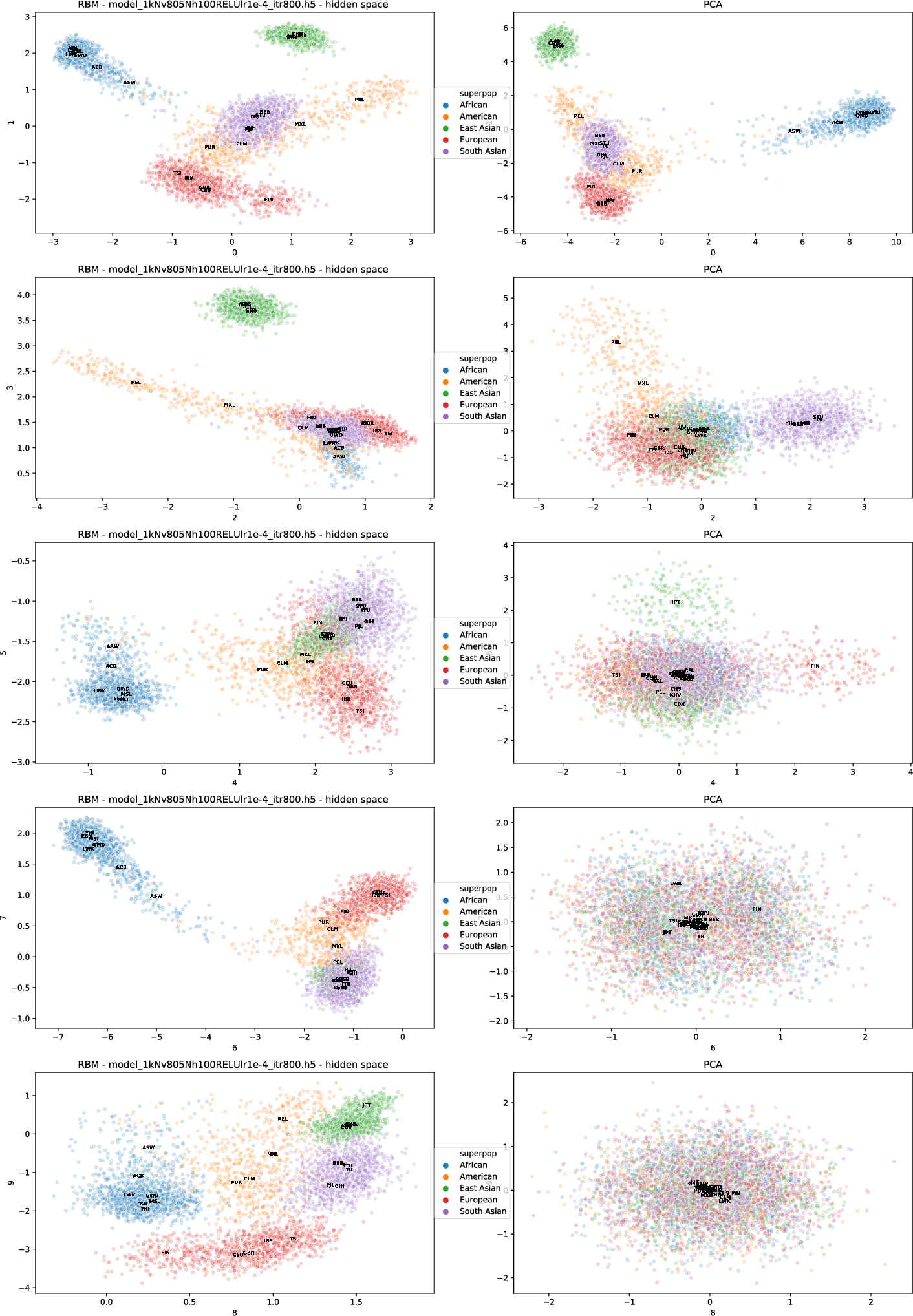

Supplement: S15 Fig — The RBM reduction was obtained by projecting the real data into the hidden space of the RBM (see Materials & Methods). Population codes are as defined by the 1000 Genomes Project. (TIF) [file pgen.1009303.s015.tif]

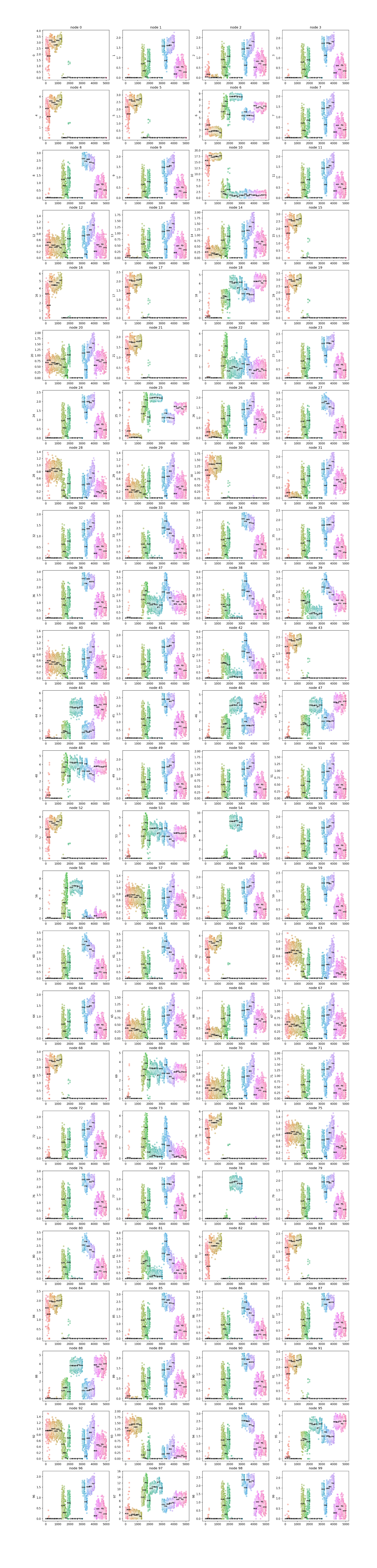

Supplement: S16 Fig — For each hidden node the X-axis corresponds to the real haplotypes and Y-axis to the activation of the node by a single haplotype. On the X-axis, haplotypes are ordered by region (Africa, America, East Asia, European, East Asia) and colored by population. Because this RBM activation function is a ReLU with threshold 0 (by design), all values are positive and a zero-value indicates that the node is not activated by a given haplotype. The ordering of nodes has no specific meaning. (TIF) [file pgen.1009303.s016.tif]

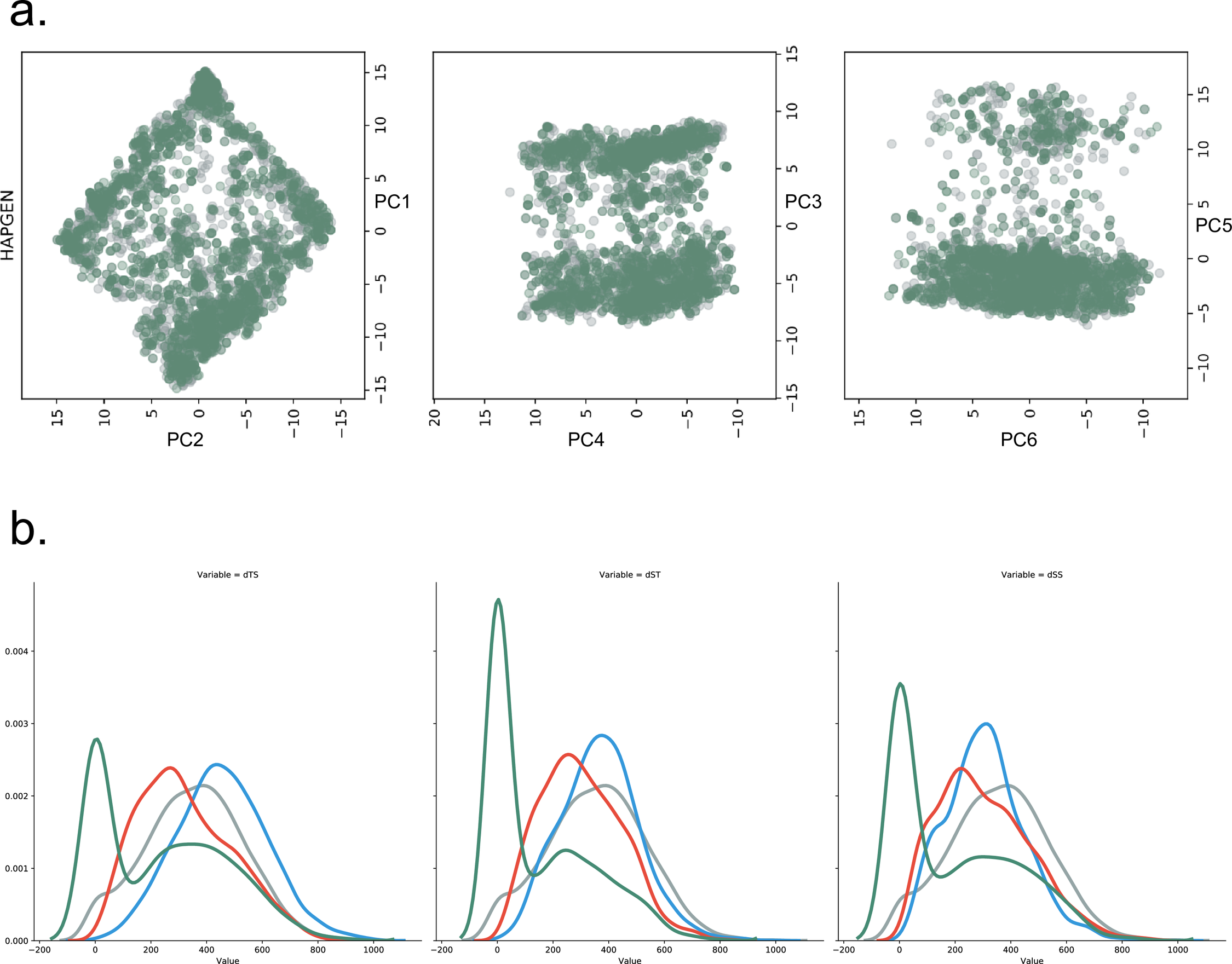

Supplement: S17 Fig — Analyses of artificial genomes generated by HAPGEN2 showing a) PCA of generated (green) performed with real Estonian genomes (grey) and b) distribution of minimum distance to the closest neighbour displaying real Estonian genomes (grey), HAPGEN2 (green), GAN (blue) and RBM (red) artificial genomes. (TIF) [file pgen.1009303.s017.tif]

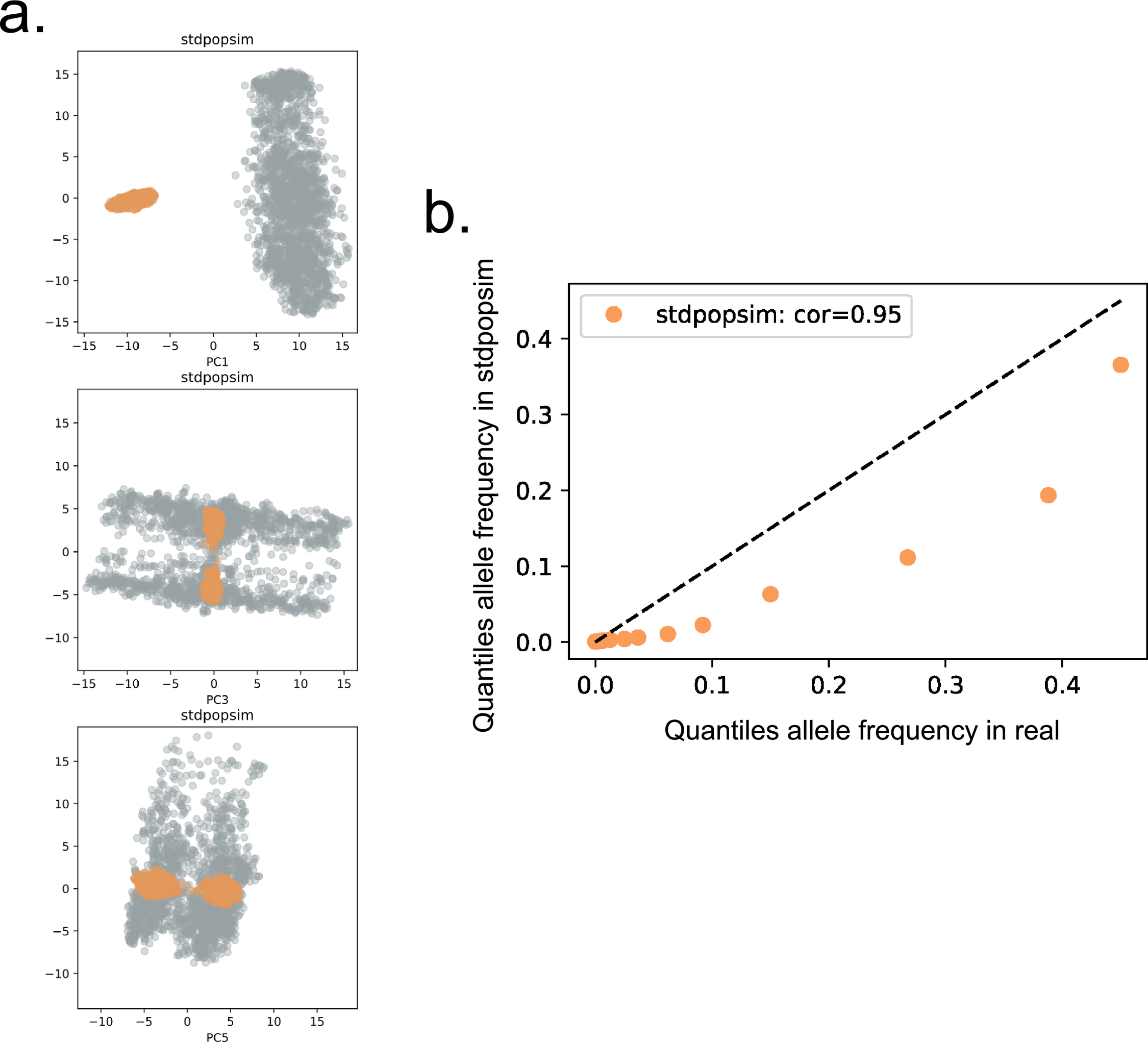

Supplement: S18 Fig — a) PCA of real (Estonian) and artificial genomes simulated via coalescent approach using stdpopsim (CEU). b) Allele frequency quantiles of real (Estonian) vs artificial genomes simulated via coalescent approach using stdpopsim (CEU). (TIF) [file pgen.1009303.s018.tif]

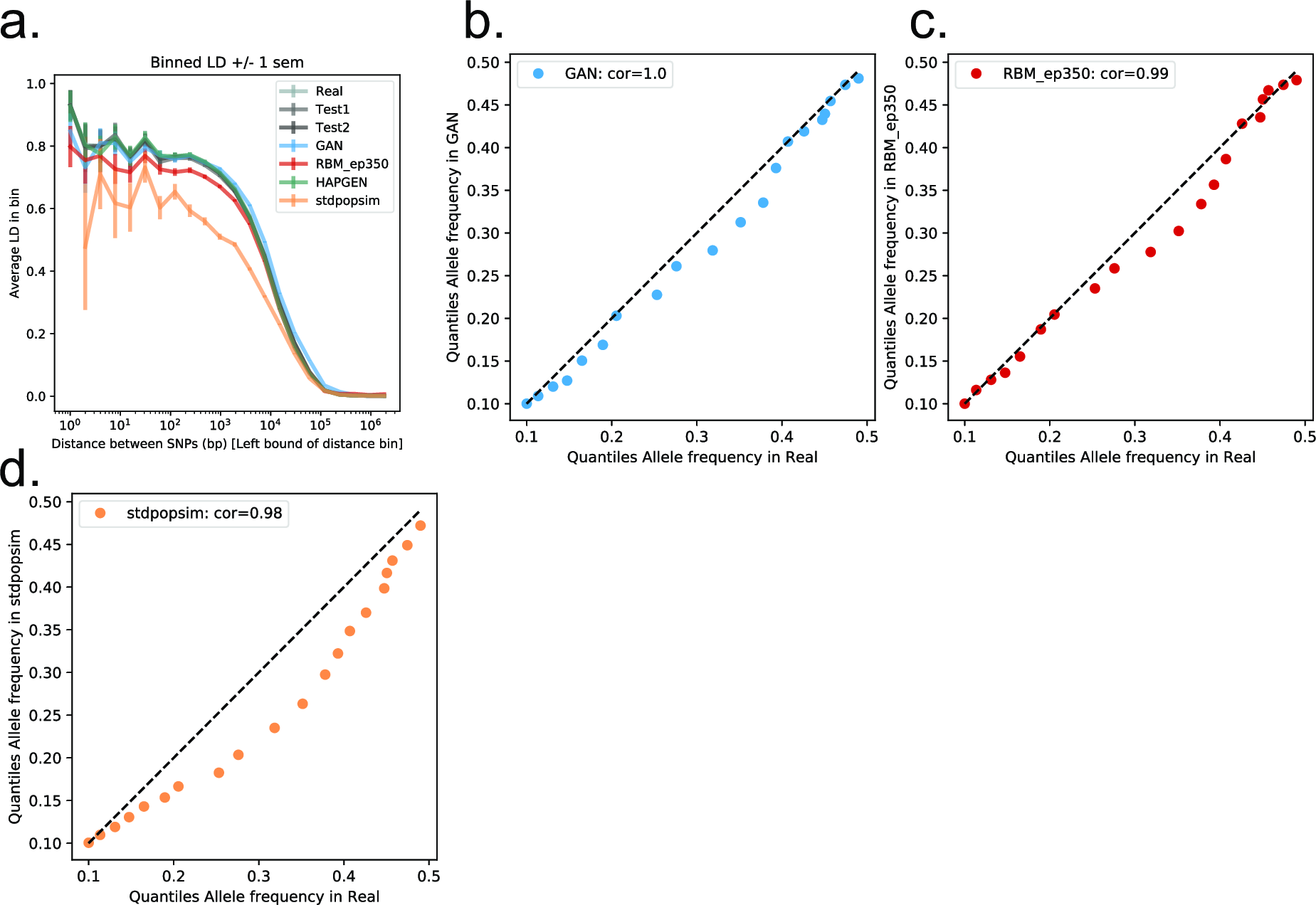

Supplement: S19 Fig — a) LD as a function of SNP distance after removing sites that are fixed in at least one dataset and removing alleles below 0.1 frequency from all datasets. Pairwise SNP distances were stratified into 50 bins and for each distance bin, the correlation was averaged over all pairs of SNPs belonging to the bin. Allele frequency quantiles of real (Estonian) vs b) GAN Estonian artificial genomes, c) RBM Estonian artificial genomes and d) artificial genomes simulated via coalescent approach using stdpopsim (CEU). (TIF) [file pgen.1009303.s019.tif]

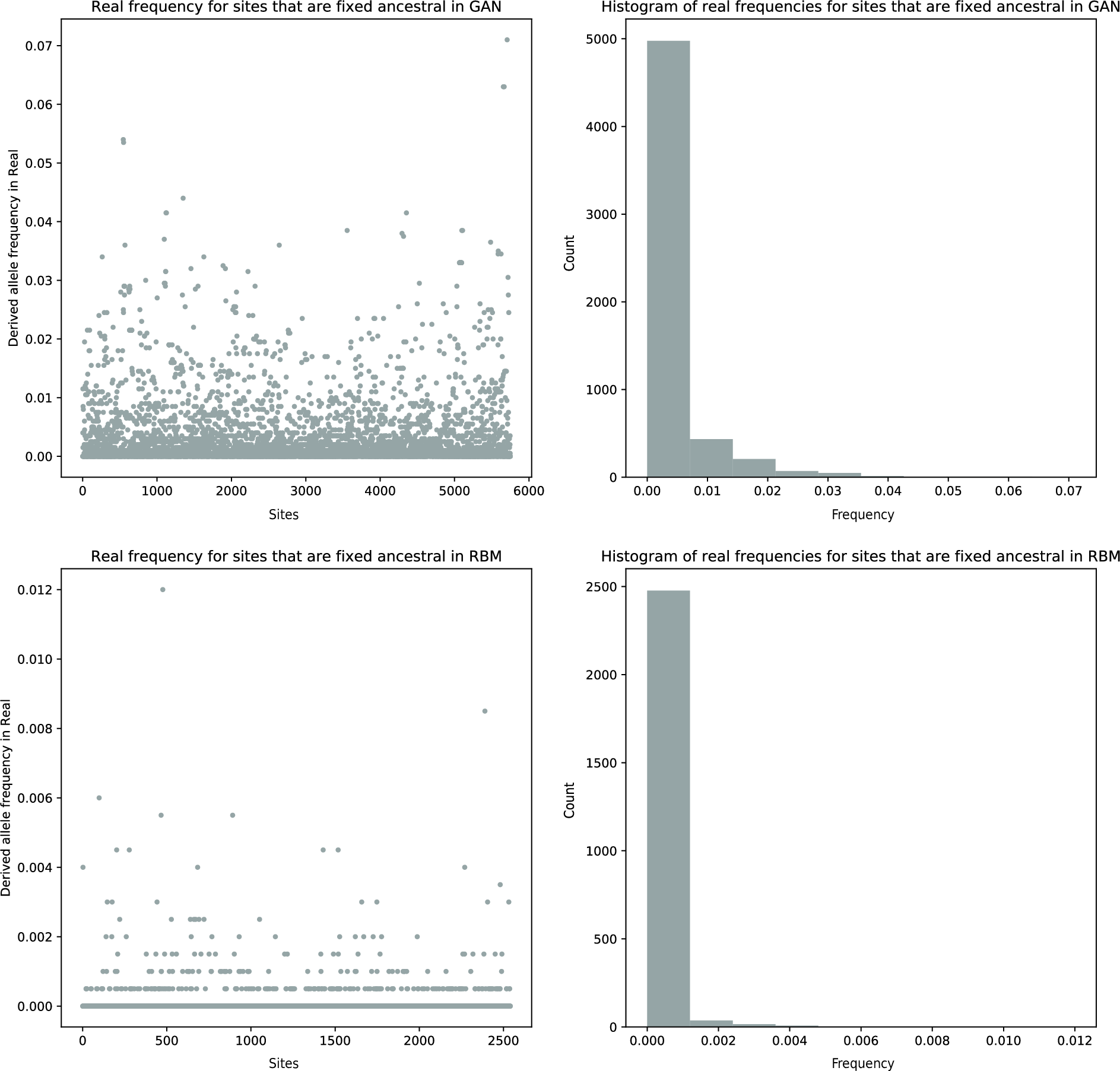

Supplement: S20 Fig — (TIF) [file pgen.1009303.s020.tif]

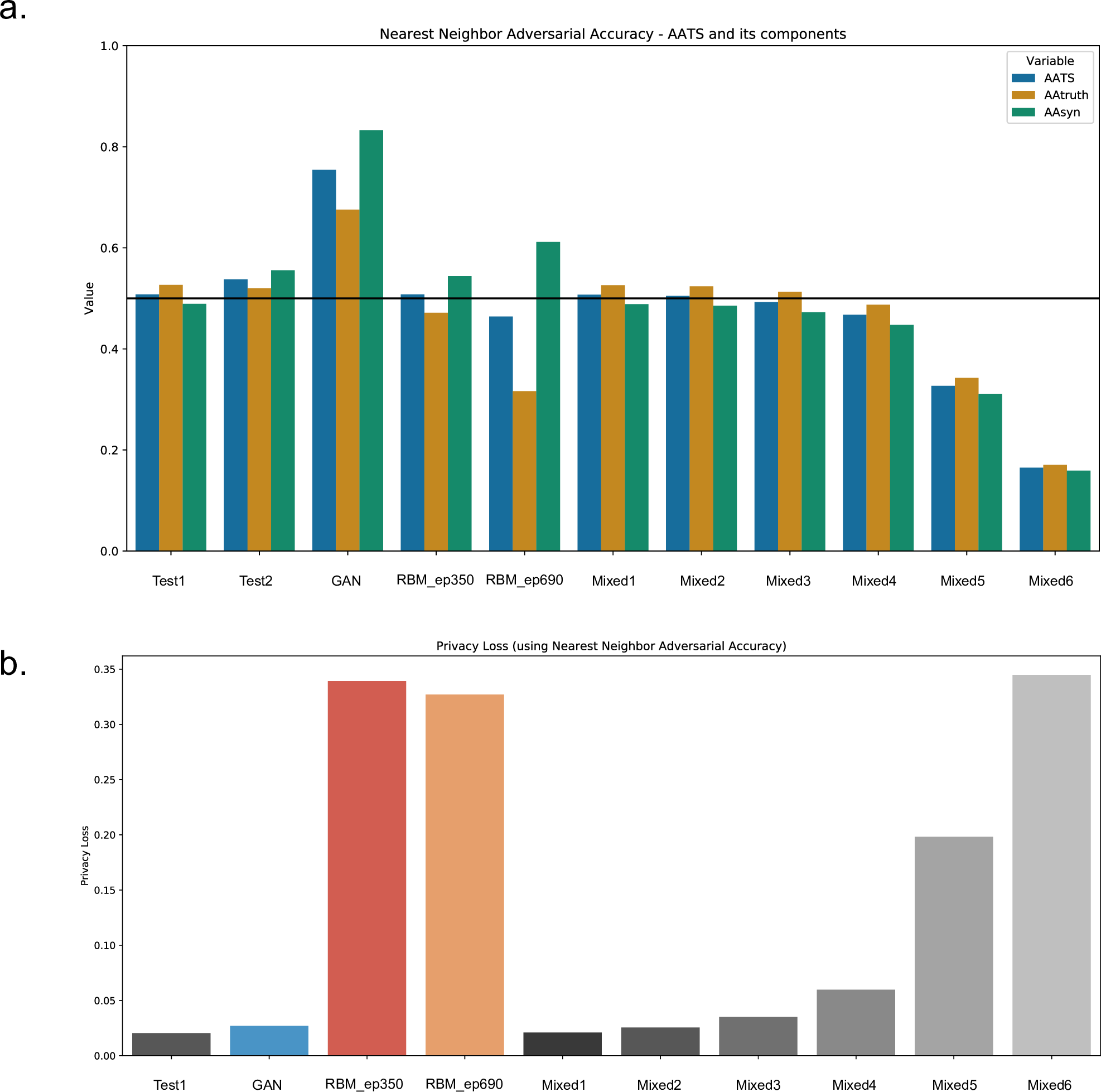

Supplement: S21 Fig — Sensitivity tests for a) AATS (scores over 0.5 indicate underfitting and below 0.5 indicate overfitting) and b) privacy scores (orange and red lines to mark the difference between RBM trained up to 350 and 690 epochs). All datasets consist of 2000 samples. Test1 and Test2 are real Estonian individuals who were not used in training. Mixed1 dataset has 1 real individual from the training dataset, Mixed2 has 10, Mixed3 has 50, Mixed4 has 100, Mixed5 has 500 and Mixed6 has 1000 individuals. (TIF) [file pgen.1009303.s021.tif]

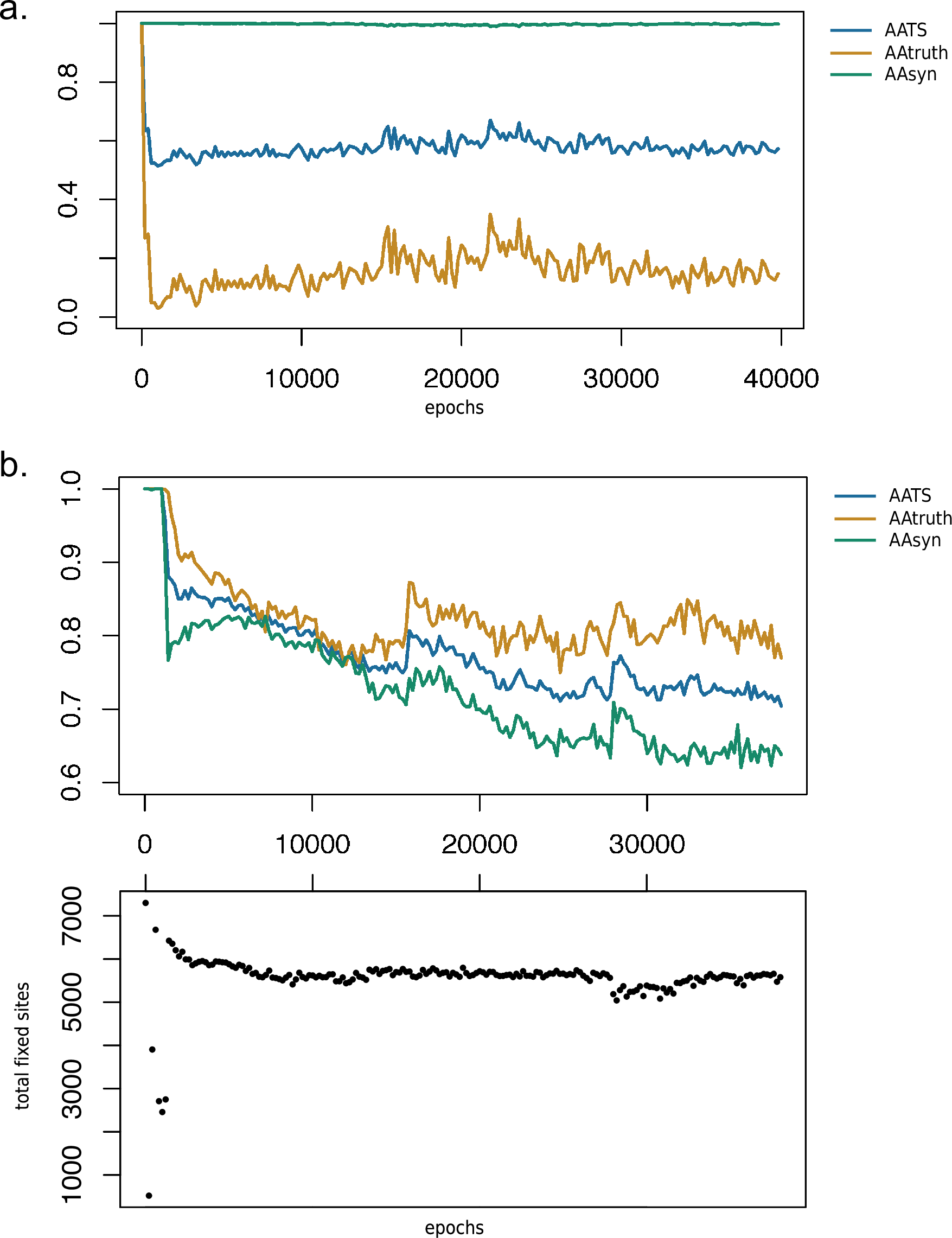

Supplement: S22 Fig — Evaluation of AATS scores of the GAN model for artificial Estonian genomes spanning a) 805 highly informative SNPs and b) dense 10K SNPs along with the total fixed sites for the outputs of epochs at 200 intervals. (TIF) [file pgen.1009303.s022.tif]

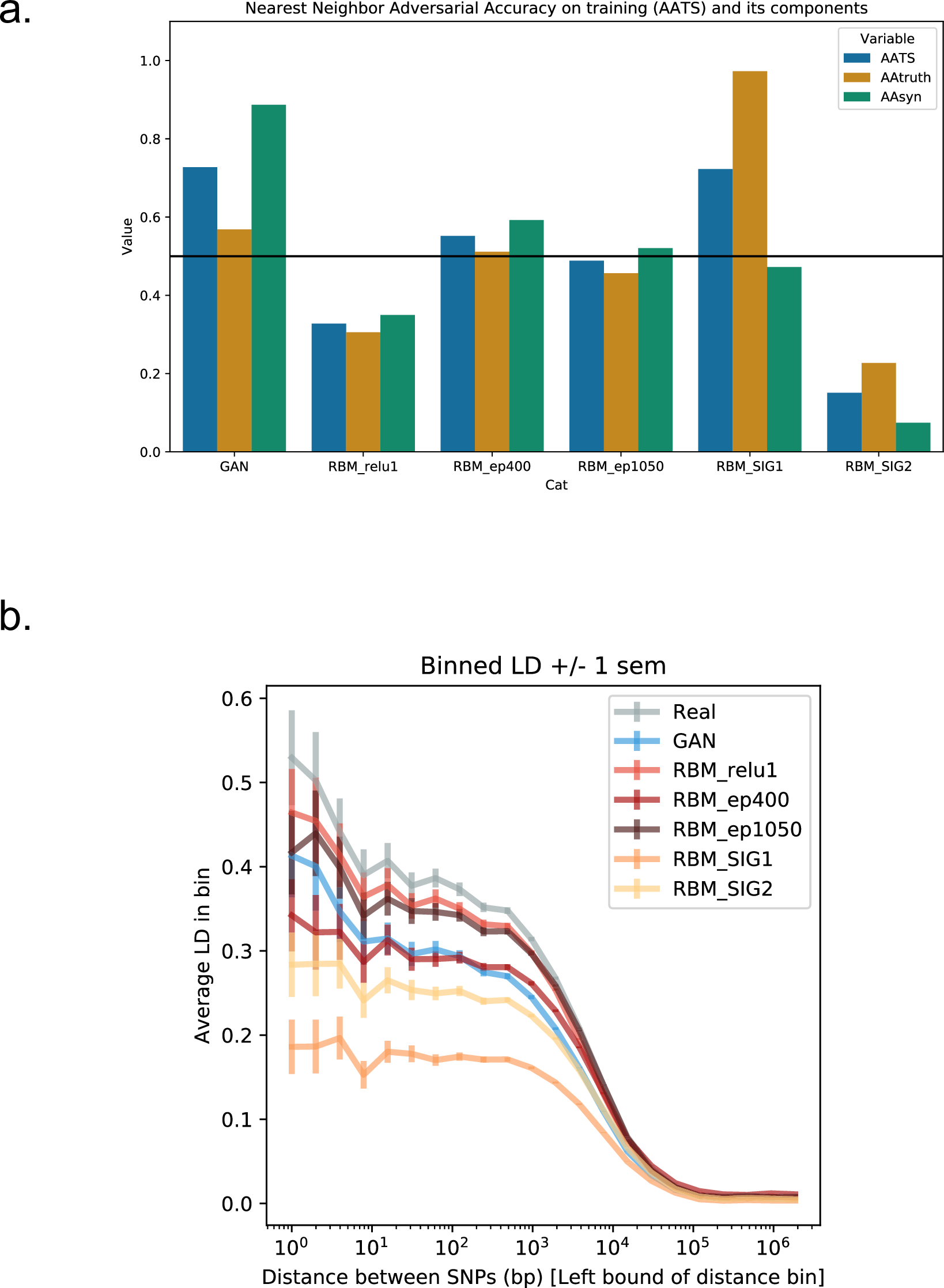

Supplement: S23 Fig — Comparison of a) AATS score and b) linkage disequilibrium of artificial genomes created via RBM model with sigmoid and ReLu activation functions. (TIF) [file pgen.1009303.s023.tif]
