# Supplementary material for: Creating artificial human genomes using generative neural networks
Source: PLoS Genet. 2021 Feb 4;17(2):e1009303. doi: 10.1371/journal.pgen.1009303 (PMC7861435; doi:10.1371/journal.pgen.1009303)
Supplement: S1 Table — Ancestral allele “A” is associated with brown eye color and derived allele “G” is associated with blue eye color phenotype. (DOCX) [file pgen.1009303.s024.docx]

| **Real** | AA | AG | GG | Total |
| --- | --- | --- | --- | --- |
| Blue | 0 | 24 | 943 | 967 |
| Brown | 41 | 615 | 302 | 958 |
| Total | 41 | 639 | 1245 | 1925 |

| **AG** | AA | AG | GG | Total |
| --- | --- | --- | --- | --- |
| Blue | 9 | 95 | 778 | 882 |
| Brown | 28 | 377 | 638 | 1043 |
| Total | 37 | 472 | 1416 | 1925 |
