# Supplementary material for: Creating artificial human genomes using generative neural networks
Source: PLoS Genet. 2021 Feb 4;17(2):e1009303. doi: 10.1371/journal.pgen.1009303 (PMC7861435; doi:10.1371/journal.pgen.1009303)
Supplement: S1 Text — (DOCX) [file pgen.1009303.s025.docx]

**Preliminary analysis on generating artificial genomes with corresponding phenotypes:**

We explored the possibility of creating AGs with unphased genotype data and recreating phenotype-genotype associations using generative models. As a proof of concept, we created GAN AGs via training on 1925 Estonian individuals with 5000 SNPs using unphased genotypes instead of haplotypes. There was an additional column in this dataset representing eye color encoded as “0” or “1” (967 blue and 958 brown eyed individuals). This region encompasses rs12913832 SNP which is highly associated with eye color phenotype. In our real genome dataset, nearly 96% of the individuals possessing at least one ancestral allele (A) have brown eye color while this percentage is 80% in GAN AGs. Similarly, 97% of all blue-eyed real individuals and 88% of the artificial ones are homozygous for the derived allele (G). No blue-eyed individuals are homozygous for the ancestral alleles in the real dataset and only 9 individuals out of the 1925 GAN AGs were homozygous ancestral with blue eyes (S1 Table). Chi-square tests based on the contingency tables were highly significant for both the real and artificial datasets (p-values < 2.2e-16 ; Supplementary Table).
