# Supplementary material for: Creating artificial human genomes using generative neural networks
Source: PLoS Genet. 2021 Feb 4;17(2):e1009303. doi: 10.1371/journal.pgen.1009303 (PMC7861435; doi:10.1371/journal.pgen.1009303)
Supplement: Attachment — Submitted filename: reviewer_response_Yelmen2020_revised.docx [file pgen.1009303.s026.docx]

We thank the editor and reviewers for all the positive feedback and suggestions and will address the criticisms below point by point. We are confident that our revised manuscript improved substantially and has benefited greatly from addressing these comments.

**Reviewer #1:**

In this manuscript Yelmen and colleagues use deep generative models to learn a distribution over real haplotype (or genotype) data such that realistic artificial haplotypes (or genotypes) may be simulated from the model.  Methodologically, I found the paper to be an interesting and technically sound proof-of-concept.  The application of these types of generative modeling approaches to statistical and population genetics is novel, and the fact that the generated data match real data so well in important ways (e.g., utility in imputation, recapitulating selection scans) is frankly surprising.  I am still not convinced about the motivation for this work, however, and a number of the numerical experiments could be further developed.  I discuss my major and minor comments in more detail below.

Major Comments:

I am not convinced by the motivation for this work, and I think that reframing the abstract, introduction, etc... might make the impact of the work more clear.  While I understand that privacy concerns mean that individual level genotype data is usually not shared with phenotype data, I find the application of AGs in these situations a bit unrealistic.  If I were running a GWAS or performing a selection scan on AGs, I could never be confident in the results, so much so that I would prefer to collaborate with someone who had access to the original individual level data just to make sure that my GWAS or selection scan results were not an artifact of the AG generation process, and I think that this would be the common sentiment among practitioners.  This substantially limits the utility of AGs.  To me, the imputation application is much more convincing because that is a concrete task that can be benchmarked and so there can be some faith in the results of imputation using AGs.

Unfortunately, I also find the imputation application to be less seemingly widely applicable -- discussing or presenting other realistic applications for which AGs might be used along these directions would go a long way toward highlighting their utility.

We thank the reviewer for their comments. We agree that the best-case scenario is to conduct research on real data. However, having a very precise proxy dataset would allow researchers to be able to test ideas without committing to full-on collaborations or projects. Easy accessibility to datasets is a problem we encounter frequently. We also believe a broader availability of genomic databases would benefit many researchers who could be struggling to set up collaborations in the first place. We have updated our manuscript to express our ideas more clearly throughout the manuscript, such as a section in Discussion part […enable preliminary genomic analyses and proof-of-concept before committing to long term application protocols and/or to facilitate future collaborations to access private datasets…].

We believe that the imputation application is very promising, but we agree that the practical applications with the current form of our method might not be easily performed due to the length limit of artificial genomes. However, we see our approach as the first step towards more practical applications with solid initial results. We updated a section in discussion to make our point more clear. [...Although being not very practical in its current form…]

We additionally discuss that AGs could simply serve as an input to downstream known operations such as forward steps of a specific evolutionary process (e.g. recent negative or positive selection, pedigree simulations, mating under specific rules leading to skewed offspring distributions or specific mate choices, complex admixture processes, ...).  This would allow testing methods inferring these processes while (a) starting from multiple variations of a real dataset (similarly to a bootstrap) rather than a single point, or (b) starting from a dataset closer to the research interest (if the original dataset was private). Initializing a simulation with real data is a procedure that is commonly used in population genetics (e.g. Martin et al 2017, Fortes-Lima et al 2019). [...They can be utilized as input for downstream operations such as forward steps of a specific…]

I was unable to find details about how the PCA and UMAP plots were made in the manuscript.  Are the generated data projected onto the PCs of the real data?  If so, how is such a projection performed in the UMAP case?  If PCA is applied independently to the generated data and real data it is surprising that they line up so well -- is there an additional scaling or rotation step involved to align the separate PCAs?

Both PCA and UMAP were performed by combining all the data and performing a single analysis (eg for Figure 1). This ensures direct comparison of the component scores. We also performed PCA on Real + one type of generated data, for example, to monitor performances during training or to compute the distance between real and generated PCA representations (see methods). We now clarified the procedure in our manuscript by adding a paragraph to Materials & Methods section. [...PCA were computed on all datasets combined…]

In Figures 2a and 3 it is difficult to assess how well the generated data is recapitulating the real data.  The results obviously look good "by eye" but it would be nice to make comparison easier.  For example, instead of plotting pairwise r^2 between SNPs in Figure 2a for the generated and real data, it might be nice to show the difference or relative difference in r^2 between the generated data and the real data.  It would also be helpful if a held out test set of real data could be compared to the original real data so that the reader might assess how substantial the deviations between the simulated and real data are.

The main purpose of Figure 2a is to show that AGs capture the LD blocks rather than to provide a quantitative comparison. We had plotted the differences in r^2 between generated and real data but it does not help visually (see an example below). We think the LD decay of Figure 2b is more informative. We now include the Pearson correlation coefficients computed from direct comparison of LD in real versus AGs for each pair of SNPs in the manuscript. Regarding Figure 3, it shows that selection peaks are present in the AGs too. Additionally we provided the correlation coefficients as statistics in the caption.

We followed the reviewer’s suggestion and split randomly the 1000Genomes dataset in two, and considered half of it as the real dataset and half as a “perfectly generated” dataset (called test) to provide reference values regarding the best achievable distances or correlations between real and generated summary statistics. We also provide the performances of the Estonian test set. This is now explained in Material and Methods and the values are integrated throughout the Results section.

Calling the simulated haplotypes/genotypes "artificial genomes" is a bit misleading as at present the simulated data represent less than a single chromosome.  Perhaps "artificial haplotype" would be more accurate.  I also find the final sentence of the discussion too sweeping -- I do not think applying generative modeling tools from machine learning to population genetic data constitutes a "forthcoming field".  Furthermore, "artificial genomics" is probably too similar to the already existing "synthetic genomics".

We agree that the term might be slightly misleading as of now but we plan to expand it to cover whole genomes in the future. We now removed the term “artificial genomics” and mention of "forthcoming field" from the manuscript.

Do the imputation results in Figure 4 depend on the number of AGs?  It would be interesting to see if increasing the number of AGs improved phasing.  It would also be interesting to compare to the case of using only AGs instead of combining the AGs with 1KG.

Using only AGs resulted in less accurate results, and we wanted to apply the suggested approach for imputing distinct populations based on Gurdasani et al. 2015 and Mitt et al. 2017 as they achieved higher scores when merging panels with 1000 Genomes. Below we also provide a figure showing the results with 20k AGs rather than 2k AGs. The performance is not significantly different (overlapping standard error of mean). We opt to not include these in the manuscript not to overwhelm readers, but we added a sentence in the manuscript (...Increasing the number of AGs did not affect the results significantly…).

Although not necessary to implement for the present manuscript, one way to scale this approach to the full genome would be to train separate GANs for each of the "approximately independent LD blocks" found by Berisa and Pickrell (Bioinformatics 2016) and then stitch the haplotypes generated in each of these regions together.

We thank the reviewer for this insight and took a note of the suggested method for future research and we agree that the suggested approach is a plausible way of producing whole genomes. We now mention it in the discussion.

There were a number of typos throughout the manuscript (e.g. "Additional 2000 Estonians were used as a test dataset").  While these certainly did not impede the communication of the science, some thorough copy-editing would improve the readability and flow of the manuscript.

We now updated the manuscript and tried our best to eliminate typos.

**Reviewer #2:**

This manuscript, "Creating Artificial Human Genomes Using Generative Neural Networks", Yelman et al., proposes a novel approach toward producing artificial/simulated genomes that is important and probably overdue. This paper takes the first step toward an ambitious goal: to simulate genomes without an explicit parameterized model while still mirroring properties of real data. Of course, we would expect the initial attempt at such an ambitious task to have flaws, and for the most part the authors have done well to highlight their methods' shortcomings and discuss how future work in this area might be able to address some of them. I particularly enjoyed the discussion of privacy matters and the need for developing better tests for ensuring that such artificial genomes cannot be used to recover potentially identifying information from the real data. However I do think there are number of critical shortcomings that were not adequately addressed, especially regarding

the purpose/utility of AGs that have no evolutionary history/ancestry, no true parameterization--indeed no parameterization at all (see below). Discussion of this should be expanded in a revision, along with changes addressing numerous other comments/questions that I raise below.

General comments/concerns:

I think the biggest shortcoming with the manuscript in its current form is that the scope of the problem is not defined. In particular, because we know nothing about the evolutionary history of these genomes (because they have none), there are many applications for which the AGs created here will not be helpful (i.e. demographic inference, detecting selection, etc). For example, if I want to test a method for finding population-specific sweeps such as XP-EHH, I have to know in which of my AG populations there has been selection and in which there has not, and this is not possible. While the authors have generated AGs parroting a specific locus under selection, such an approach will be biased (one can only do this for "known" sweeps which are few and far between, and will be those with extraordinarily strong selection) and of debatable utility. I think the manuscript needs an extensive, detailed discussion of which tasks can and cannot be addressed using their method, focusing on current limitations and discussing whether any future developments could address these.

We thank the reviewer for the comments. It is true that we do not know the evolutionary history of AGs and in its current form, they cannot be used for applications which require a priori knowledge of the ground truth. AGs can be seen as surrogates of real genomes. Testing a method as suggested would not be possible with real genomes either, since the ground truth is not known. Conventional coalescent simulations might be a choice for such tests. However, it should be possible in the future to use conditional GAN/RBMs and to train them on coalescent/forward simulated datasets to create AGs based on specific parameters. More simply, AGs could also serve as an input to downstream known operations such as forward steps of a specific evolutionary process (e.g. recent negative or positive selection, pedigree simulations, mating under specific rules leading to skewed offspring distributions or specific mate choices, complex admixture processes, ...).  This would allow testing methods inferring these processes while (a) starting from multiple variations of a real dataset (similarly to a bootstrap) rather than a single point, or (b) starting from a dataset closer to the research interest (if the original dataset was private). Initializing a simulation with real data is a procedure that is commonly used in population genetics (e.g. Martin et al 2017, Fortes-Lima et al 2019). We now added these ideas in the manuscript (...They can be utilized as input for downstream operations such as forward steps of a specific…)

On the other hand, AGs could be used in reference databases for imputation and ancestry analyses, serve as outliers in selection scans or be used as realistic toy datasets that do not break any privacy.  The perspective, once the-state-of-the art is adequate, is that AGs could help in most analyses where the real, but private, genomic dataset would have provided useful information. We now updated our discussion to include these points.

We would also like to clarify that the analysis was not supervised to “parrot a specific locus under selection”, all signals of selection contained in a region could in theory be captured and reproduced, although weak signals could indeed be lost.

On a related note, beyond the evidence of overfitting presented by the authors, there may be some other manners in which the AGs are "too much" of a copy of the real data? For example, the AGs seem similar to the point of mimicking the spatial arrangement of "ancestries" along the chromosome. Is this a desirable characteristic? Or would we instead want the mosaic of ancestries to be drawn from the same distribution of that of the real data, rather than perfectly matching that of the input.

The Chromopainter figure (Supplementary Figure 10?) shows that there is variation on how the genomes are painted. So we do observe a variation of the arrangement of the ancestries and agree that it is a desirable characteristic. What we believe to be important though is that the Estonian AGs should be mostly painted by European genomes as it is the case for real Estonian genomes. If Estonian AGs would be mostly painted by anything other than European genomes, that would be an undesirable outcome. As noted in the manuscript, the “mosaic distribution” of the GAN does not perfectly match the real one as there was an excess of short chunks in AGs compared to the real genomes. This is now clarified in the text (..However, the majority of the AGs produced via GAN model displayed an excess of short chunks when painted against 1000 Genomes individuals, whereas we do not observe this for RBM AGs..)

In the current version of the authors' tools, are we limited to output sample sizes less than or equal to the training set sample size? Or could the methods generate larger numbers of AGs (and if so, how would it perform under this scenario)?

Once the model is trained, it is possible to generate virtually unlimited number of AGs. For the GAN this consists in a forward pass in the trained generator network, while for the RBM it requires sampling from a MCMC that has already converged, with steps ensuring independence of the samples. Thus, the RBM generating process is longer than the GAN but in both cases nothing prevents from generating more individuals than the training set size. We now added a sentence in the methods. (...After the training is complete, it is possible to generate as many AGs as desired...)

There are several places in the results where the authors are referring to the population of origin of particular artificial genomes. How is this information known? Is it preserved in the ordering of chromosomes in both the real and artificial data? Or is it encoded some other way, or simply inferred after the fact via PCA, etc?

This information is not known and we did not try to infer it. It is only known for AGs generated using uniquely Estonian genomes. We failed to find any such part in our results which we refer to a specific population of origin. A modification of the method would allow exploiting these labels (as mentioned in the discussion), however showing that we can train accurate generative models without prior knowledge on the dataset is a more difficult task and demonstrates the strength of the method.

In my view it is not acceptable to keep the code under wraps prior to publication. At the very least, all code related to the architecture, training, and testing/application of GANs/RBMs should be made available to reviewers prior to acceptance, but I defer to the editors here.

We agree with you and apologize, we now made the code publicly available at “<https://gitlab.inria.fr/ml_genetics/public/artificial_genomes>”.

Re: Linking genotypes with phenotypes: I am not sure what the utility of this is. Is it to give us an alternative way of simulating genotype-phenotype associations? If so this will be biased because we can only use it in cases based on real data where the genotype-phenotype association is known. So it will have very little utility for assessing power. Also, will the "effect size" differ between the real and artificial data? Do we have any way of knowing this? More discussion of these issues is needed.

Related to your first comment, it is important to note that we do not plan to directly use AGs to test case-control studies. The idea behind our approach is that we do not need to know the association, we only need datasets with genomes and phenotypes of real people and use these without any prior knowledge to generate AGs. We performed this application to show that (i) not only haploid but also diploid type of data can be generated and (ii) genotype-phenotype associations can be captured. Once the generative models scale up to the entire genome, they could be extended to include more phenotypes.

The authors seem to favor the performance and feature representation of the RBM over the GAN, and while I agree that by some measures the RBM seems preferable, it has numerous drawbacks (especially overfitting) and some of the comparisons performed come across as subjective and unconvincing. For example:

        -Line 128: Referring to Supplementary Figure 4, the authors say that "Especially RBM excels at replicating the real peaks", but there is no quantitative comparison and to my eye perhaps the GAN is doing as nearly as well.

We clarified our remark that concerns the modes of the pairwise distribution and added the distances computed between the overall distributions (real vs generated).

        -PCA is shown in Figure 1, and there the RBM seems to better reflect the clustering the data than does the GAN. However, one could argue that this is reversed in Supplementary Figure 3 (UMAP results).

We now include distances between the real and generated PCA or UMAP representations.

        -Lines 141-143 and Supplementary Figure 7: The authors correctly point out that the GAN performs poorly for low frequency alleles, but here the RBM is all over the place all across the frequency spectrum.

This sentence now reads “[...] we see that especially GAN performs poorly for low frequency alleles, due to a lack of representation of these alleles in the AGs whereas RBM seems to have wider distribution over all frequencies” and we included measures of correlation between real and generated frequencies.

More generally, we updated the results to provide multiple scores allowing a direct comparison between models: Wasserstein distance between 1D or 2D distributions (pairwise difference distributions and PCAs), as well as the Pearson correlations coefficients  for all direct comparisons (eg frequency at site i in real versus artificial, LD at pair (i,j) for real versus artificial 3-point correlations at triplet (i,j,k) for real versus artificial).

-Line 208: Despite it's proclivity to overfit, the RBM did not capture the genotype-phenotype association of the GAN. This is surprising because this is similar to capturing LD between polymorphisms which the RBM seems to do quite well, and perhaps this fact should be noted (either there or in the Discussion where the authors mention possible improvements to the RBM for this task).

We already speculated and offered a possible solution in the manuscript about this in discussion [...We believe that it is because the encoding of the…].

Specific comments:

-Abstract, lines 24-25: It is not clear what the authors mean by "learn the high dimensional distributions of real genomic datasets."

We changed the sentence to: “In this study, we demonstrated that deep generative adversarial networks (GANs) and restricted Boltzmann machines (RBMs) can be trained to learn the complex distributions of real genomic datasets [...]“

-Supplementary text on RBMs: I do not see why this relatively short section shouldn't be an additional subsection within the methods rather than supplementary text.

Due to manuscripts’ size limitation, we opt to keep it supplementary.

-Supplementary Figure 4: What is the purpose of the right panel (comparing each AG dataset to haplotypes from the real dataset)? I see that these results look more similar to the Real vs. Real comparison, as one might expect given that in each comparison in this panel one of the haplotypes is always real. Please explain what the reader supposed to learn from this panel (it is not mentioned in the text as far as I can tell).

The distribution of pairwise distances within a dataset are informative about the hidden structure of the data. On the other hand the distribution of distance between real and artificial genomes can be informative on phenomenon of overfitting (distances smaller than expected, i.e. global shift of the distribution towards the left or emergence of a mode at shorter distances; copying of individuals directly from the source leading to pairwise distances of 0) and underfitting (distances larger than expected). This inspection is then pushed further using the minimal distances distributions and summarized by the AATS scores. We now updated that part in the manuscript.

-Supplementary Figure 7: It is not clear how the authors are generating this plot or why they visualizing in this way rather than simply comparing the allele frequency spectra between the Real, GAN, and RBM datasets. Some explanation is required.

In this plot, x axis is the allele frequencies in the real dataset and y axis is the allele frequencies in the AG datasets. We decided to use this plot because the deviations for low frequency alleles in GAN dataset was clearly visible. This deviation is also mentioned in the manuscript. Additionally, the plot shows the correspondence of SNPs between real and artificial genomes, which is an important feature absent in genomes simulated with the coalescent approach.

-Supplementary Figure 8: I think the purpose for this comparison could be more clearly articulated at this point in the text. The point is to see whether AG individuals look too much like those from the real data (i.e. whether the GAN/RBM methods are merely copying real genomes rather than drawing individuals from the same distribution), right?  This is discussed more later on but some readers might be confused when reaching lines 143-147.

We added additional explanation in that part. [...On the other hand, the distribution of the distance of real genomes to the closest AG neighbour shows that …]

-Supplementary Figure 10: The legend for this figure does not explain the difference between the left and right panels.

For each dataset (a, b, c), two painted haplotypes are shown (left and right panels). These haplotypes were picked to illustrate different AG behaviour (for example fragmented versus smooth mosaic of ancestries). We now updated the legend.

-Lines 180-182: I wonder how the RBM with reduced (but still substantial privacy loss) performs according to the other analysis done to assess the fit of the AGs to the real data. I would add PCA/UMAP/pairwise haplotype distance distributions for this set as a supplementary figure.

We picked a dataset generated with the RBM alternative scheme that has a privacy loss of 0.08 for the training set and 0.1 for the sampling set. We inspected its various representations. The correlation between generated and true allele frequencies was 0.92 (instead of 0.95 for the previous RBM and 0.98 for GAN), the correlation for LD values was 0.97 (RBM:0.98, GAN:0.97), the 2D-Wasserstein distance for the PCA representations was  0.026 (RBM: 0.006, GAN: 0.011, RBM sampling initialized randomly: 0.339), the Wasserstein distance for the pairwise distribution was 97 (RBM: 26, GAN: 227, RBM sampling initialized randomly: 689). We thus observe a small decrease in accuracy for certain summary statistics although the performances remain satisfactory while the privacy loss was reduced from 0.34 for the regular training scheme to at most 0.1 with the alternative scheme. This is now summarized in the text [… A dataset produced by this alternative scheme had only a slight decrease ...]

-Selection tests section, somewhat echoing my general concern above here: It is unclear what the purpose of this small section is. The authors show that tests for population-specific tests for selection produce outliers in both real data and in AGs. How does this imply that "AGs can also be used for selection tests?" Used in what way? Also, the last sentence in this section is incomplete, and its meaning is unclear. This section needs to be re-written for clarity (i.e. what is the precise goal, and how do the results demonstrate that this goal is achieved?).

The main premise of our study is that AGs are reliable alternatives to real genomes. We want to emphasize that using real genomes will always be better. However, AGs are the second best option to run some tests if the real genomes are not accessible or if one needs to explore ideas without committing to long-term data access protocols or collaborations that might be difficult to set up. We updated “selection tests” and discussion sections to make it clearer.

Lines 235-238: Although the authors have just asserted that the RBM representation differs from that produced by PCA, the description of the first two components given here also matches the PCA results nearly perfectly, and this should be noted. For lower-rank components there are substantial differences however.

We clarified the resemblance and difference between these two representations in the text. Supplementary Figure 15 helps following the rationale. This section now reads as: […although the general structure identified by the two lower rank components is highly similar…]

Line 252-254: "The minimum required number of individuals for training is highly variable". I think it would be worthwhile sharing some more information here. What is meant by "minimum number"? Variable across what? How variable is "highly variable"?

By minimum number of individuals for training, we mean the number that enabled us to train a generative model without failure (such as mode collapse) and that converges to a possible ideal mode which correlates with real data.

The number of examples required to train a generative model is known to depend on the unknown dimension of the data to generate. It should at the very least be larger than this unknown dimension. Since this unknown dimension varies with the population(s) and type of data (e.g. highly differentiated unlinked SNPs, fine scale local variation, ...) it would require training under multiple scenarios (different population, varying chunk size, varying sample size) to be able to reply precisely. A very large number of experiments (multiple training under the same scenario) would be needed to provide an answer and each experiment is computationally intensive. Furthermore, this result would not be applicable to a new dataset. We now clarified the part. For direct application, we thus suggest that it is more valuable to investigate the performances directly on the dataset of interest.

We clarified this part [… depending on the unknown dimension of the dataset, which is linked to the type of data to be generated and the population(s) ...]

Lines 345-346: "...the forthcoming field which we would like to name artificial genomics." Although this is important, innovative work, I do not agree that this represents a new field with its own name. The generation of artificial genomes has been around for a long time, though typically through coalescent or forward simulations of course.

We removed the term.

Line 394: "Therefore, we stopped training based on coherent PCA results of AGs with real genomes." Please give more detail here.

We stopped the training based on coherent PCA results of AGs combined with real genomes performed at 200 epoch intervals as it is now included in manuscript. “Coherence” here means that PCA results are examined to eliminate poor candidates (characterized by real and generated data being projected to different directions of the PCA space). This provides an efficient criterion since GAN models might be blocked in local optima with very poor outcomes and metrics to assess performance are highly variable and might not be informative (Borji 2018), especially in case of genomes that need to make sense biologically.

Line 444: Please explain precisely what is meant by "its probability given the previous sequence window".

We now updated the part. “At the initial position the allele was set by drawing from a Bernoulli distribution parameterized with the real frequency. At a given position *p* the allele *h_p_* was drawn in *{0,1}* according to its probability given the previous sequence window *of size w,* *P(h_p_ | h_p-w_, …, h_p-1_).* This probability is computed from the observed haplotype frequencies in real data.”

**Reviewer #3:**

In this manuscript the authors explore the use of generative deep learning methods (GANs and RBMs) for the generation of simulated, or as the authors say, artificial genomes. The idea is to train a generative model on an empirical dataset, e.g. 1000 Genomes, and then with a trained model in hand, walk the learned latent space to output simulated samples. The authors compare generative methods, then examine the output from those with a battery of population genetic statistics/analyses.

The idea of using generative deep learning methods has taken off over the past decade in other fields thus the application to genetic data is both timely and reasonable. What the authors have done here is a nice step in that direction, however I have a number of comments and criticisms that I would like to see addressed.

Major points:

1) What the authors are doing here amounts to simulation of genomes from a black box. As the authors know, a large portion of the field is devoted to inferring demographic models from genetic data that themselves are ground-truthed via simulation. It strikes me that the authors are not using a sensible baseline comparison in this paper. Currently they compare their methods to a Bernoulli and Markov model, both of which are rather ad hoc. A more appropriate comparison would be to parametric simulation of the population data (e.g. from the coalescent). A relatively simple thing might be to pick one of the models implemented in the stdpopsim library (https://www.biorxiv.org/content/10.1101/2019.12.20.885129v1), train a GAN and an RBM on the original data, and then compare the coalescent simulations (truly a generative model) to these new methods.

Such comparison to simulations should extend not only to PCA type visualization comparisons, but also to patterns of LD, sweep signatures, and imputation. Further a run time comparison would be appropriate, and should heavily favor the trained DL methods.

We thank the reviewer for their comments. Coalescent simulations create parameter-based genomes which mimic certain qualities of real genomes but a position by position comparison is not possible. There is no direct correspondence between the real SNPs and the ones in the coalescent simulations. In other words, simulated genomes exist in another space. For instance, if you try to perform a PCA with real CEU and CEU simulated with coalescent approach, there will be two very distinct clusters. On the other hand, AGs are fully comparable to real genomes and they exist in the same space. This means that it is not possible to directly merge real genomes with genomes generated by a coalescent (or forward) simulator. This impedes the use of coalescent-based artificial genomes for imputing real genomes or serving as reference in selection scans with real genomes (for example). Therefore, it is difficult to make a fair comparison of these two methods.

Additionally, the resolution of coalescent simulations is limited to broader population definitions. As an example, it would be very difficult to simulate Estonian genomes because of the unknown/subtle evolutionary history of such populations still understudied or only studied very recently. It is also fairly difficult to have a parametrized model for 26 different interacting populations such as in the 1000Genomes. This is exemplified by the fact that most demographic inference studies focus on studying either many populations independently or only a few populations simultaneously (i.e. with the full interaction model consisting in dates of splits, admixture and migration, together with population size changes), the classical example being one African population, one Asian, one European and one admixed population. Stdpopsim for example has no proper model for the full 1000Genomes evolutionary history.

However, we did our best to make such a comparison through the suggested approach. We picked Gutterkunst 2009 model via stdpopsim and simulated CEU individuals and are now providing some comparisons and discussion in the manuscript (section “Comparison with alternative generative models”, Discussion, Supplementary material), and we summarize below the findings.

After encoding all datasets into minor versus major alleles, the 2D-Wasserstein distances between real and generated PCA representations are the following: GAN: 0.040, RBM: 0.003, stdpopsim: 0.474, with the coalescent and real data being projected to different directions (SM Figure xx). The SNPs cannot be compared directly as confirmed by the correlation between real and stdpopsim allele frequencies (0.02 instead of 0.96 and 0.92 for GAN and RBM) or between real and stdpopsim LD measures (0.17 instead of 0.95 for GAN and 0.98 for RBM). Instead, we compared the quantiles of the allele frequency distribution and those are clearly underestimated by stdpopsim (Supplementary Figure 18b). This bias is partly due to preprocessing steps of real data (e.g. filtering from various bioinformatic pipelines) that cannot easily be reproduced with coalescent simulators. Hence, we filtered all datasets (real and generated) to remove SNPs with minor allele frequency below 10%. For these new datasets there is still no one-to-one correspondence between SNPs, so the spaces of stdpopsim and real data are obviously still different. This is a main barrier to using coalescent simulation as artificial genomes for the applications we propose. Furthermore, the LD block pattern revealed by stdpopsim differs strongly from the real one (figure below). stdpopsim is also unable to reproduce the exact decay of LD with distance and underestimates the level of LD observed in real data, while other generative models captured both the short range and long range correlation patterns between SNPs (Supplementary Figure 19a).

We hope this analysis and additional explanation made clear why, despite having lots of advantages and being widely used in population genetics, coalescent/forward simulators are not natural candidates for the use case proposed in this study.

2) The results section need to be more quantitative somehow. For instance on L122 the authors say that the “All major modes… are well represented.” Can the authors report something like loss here? Which model fits the data better? It’s not clear. The same is true for the following sentence L123—it’s not clear which method is performing best and in UMAP space the GAN and RBM don’t seem to be fitting that well. Can you make a quantitative statement here? Again true for the comparisons of pairwise differences (SFig.4)—can you report KL distance or similar? As noted in comment 1— coalescent simulations here seem to be the logical baseline. Throughout the paper the authors should add numeric comparisons of the fits they are achieving.

We updated the results to provide multiple scores allowing a direct comparison between models: Wasserstein distance between 1D or 2D distributions (for the pairwise difference distributions and PCAs), as well as the Pearson correlations coefficients for all direct comparisons (eg frequency at site i in real versus artificial, LD at pair (i,j) for real versus artificial 3-point correlations at triplet (i,j,k) for real versus artificial).

We also added a reference for these comparisons. For that we split randomly the 1000Genomes dataset in two and considered half of it as the real dataset and half as a “perfectly generated” dataset (called test) to provide reference values regarding the best achievable distances or correlations between real and generated summary statistics. We also provide the performances of the Estonian test set. This is now explained in Materials & Methods and the values are integrated throughout the Results section.

3) With respect to the LD analysis (Lines 149-163) I have a few points that follow from comment 2. First it’s not at all clear that the haplostrips are indistinguishable by eye. This is a very hard figure to read and a qualitative comment. Second I disagree with the statement that “RBM AGs were nearly indistinguishable form real genomes” in SFig10. They look quite different to me. Again this could be resolved with a quantitative statement about similarity.

We agree that haplostrips figure is difficult to read but we merely wanted to show we do not see 3 different blocks for real, GAN and RBM AGs and we now clarified this idea in the text [...We did not observe separate clustering of real and artificial genomes with Haplostrips…].

Regarding the second point, Chromopainter is a widely used population genetics tool to visualize how the genomes are painted based on ancestry. With that statement, we meant to say we do not see excess of short ancestry chunks as we observe for GAN AGs. There is variation among real genomes too. Here the goal is not to do a one to one correspondence between an artificial genome and a real genome because the artificial genome does not mimic one real individual in particular (it is only sampled from a similar distribution than the real dataset distribution). Our point was to show the prevalence of European ancestry and undisturbed chunks. We now rephrase that sentence to make it clearer and provided average European chunk lengths for the painting.

4) The early stopping mentioned by the authors on L182 seems fine but what do the genotypes generated by the trained model look like at this point? I’d imagine they look a bit less like the real thing.

We picked a dataset generated with the RBM alternative scheme that has a privacy loss of 0.08 for the training set and 0.1 for the sampling set. We inspected its various representations. The correlation between generated and true allele frequencies was 0.92 (instead of 0.95 for the previous RBM and 0.98 for GAN), the correlation for LD values was 0.97 (RBM:0.98, GAN:0.97), the 2D-Wasserstein distance for the PCA representations was  0.026 (RBM: 0.006, GAN: 0.011, RBM sampling initialized randomly: 0.339), the Wasserstein distance for the pairwise distribution was 97 (RBM: 26, GAN: 227, RBM sampling initialized randomly: 689). We thus observe a small decrease in accuracy for certain summary statistics although the performances remain satisfactory while the privacy loss was reduced from 0.34 for the regular training scheme to at most 0.1 with the alternative scheme. This is summarized in the text [… A dataset produced by this alternative scheme had only a slight decrease ...]

5) The genotype to phenotype analysis is so minimal that it doesn’t add much as currently presented. Given the phenotype and the effect size of the SNP it basically collapses to an imputation problem. At a minimum I’d have the authors show performance with SNPs of various effect sizes, as well as a polygenic trait. The authors could similarly just cut this section of the paper and I’d be happy.

We agree with the simplicity of the approach but as we mentioned in our manuscript, this is performed as a simple proof of concept and additionally, to show that diploid data structures can be learned too. We believe it has much potential in the future so we opt to keep this part as it can inspire others to specifically investigate this point.

6) Imputation section— I’m skeptical of the utility here. It’s not clear to me how much of the small performance boost at low MAF is just due to increasing the sample size of the imputation panel. The authors should explore this by doing something like the following: take an empirical imputation panel and cut it in half, now compare the performance increase by adding back to the imputation panel the real data vs. the data simulated via the RBM/GAN. In addition I’d be curious how data simulated under the coalescent might aid in imputation. Further all of the imputation that is shown in the paper is in-sample—what happens to out of sample imputation using simulated genomes?

We would like to emphasize that all imputation panels except for the 1kG have the same size (1000 Genomes + 2000 haplotypes). Additionally, we provide below results with more GAN AGs (20,000 instead of 2,000), which shows no significant change in performance (overlapping standard errors of the mean). Therefore, increasing the sample size does not systematically increase the performance.

We also checked imputation with a more distant population and used African AGs as an addition to the 1000 Genomes panel. African AG added panel performed slightly better than only 1000 Genomes and it performs worse than Estonian AGs + 1000 Genomes panel for the lowest MAF bin confirming that the gain in performance using Estonian GAN AGs is not due to sample size. We add the figure below but opt to not include it in the paper to not overwhelm the readers.

Regarding the use of coalescent simulations for imputation, we would like to clarify again that there is no SNP to SNP correspondence in genomes simulated with the coalescent approach (they exist in a different space). This in fact, is a major difference, and advantage here, of our approach. We do not think that coalescent simulations can be used for imputation of real genomes.

7) With respect to scans for selection it’s unclear what this shows other than that the RBM/GANs are learning some local representation of allele frequencies and LD (to greater or lesser extent). Given the x axis scale on Figure 3 is 1e8 bp- I imagine these are stitched together predictions from 10Kb windows or similar? Further I don’t really agree with the statement on Line 328 that “scans for detecting selection are another promising use case”—how so? No new information about selection is gleaned here.

We now updated the selection part and discussion. Overall, one major possible utility of our approach is to provide high fidelity approximations to real genomes. There is new information compared to not having a specific real dataset at all. For example, let us suppose that a researcher has data on population X and is interested in local adaptation, useful AGs could be the public version of a private dataset of the closest neighbour of population X.

If AGs, combined or not with real genomes, capture selection signals, a researcher can perform basic selection tests on AGs before committing to a long application/collaboration procedure for a certain database. Also, values at x axis of Figure 3a are exact positions. Figure 3b was calculated based on windows as explained in Material & Methods.

8)      Not enough details are given in the methods with respect to the network architecture of the GAN. I’d suggest at least including a diagram of the network in the supplement. The same is true for the RBM.

The Material and Method section describes for all networks: the total number of layers, the nature and dimension of each layer, the learning rate, the type of loss, the optimization method and parameters, the batch size and number of epochs. The full implementations of GAN and RBM are now available on the gitlab repository at “<https://gitlab.inria.fr/ml_genetics/public/artificial_genomes>”.

9)      No code is shared! To really evaluate this the code will need to be distributed. As is there is no implementation and that is a real shame.

We agree and now provide the full code publicly available at “<https://gitlab.inria.fr/ml_genetics/public/artificial_genomes>”.

10) Lastly coining the term “Artificial genomics” as done in the last paragraph is completely unnescessary. We don’t need more jargon and we already have ways of generating fake genomes via simulation. Moreover the authors can’t even generate fake genomes with their methods—only fake windows of genomes.

We now removed the term “artificial genomics”.

Minor points:

1)      Fig2A – perhaps add label for “real” vs “synthetic”

We now updated the figure.

2)      Line 186—what does “homogeneously dispersed” mean?

It means that the selected SNPs span the whole chromosome with a similar distance between each SNP. We now made it more clear.

3)      Line 252—I would be curious to see these results on number of individuals vs training success

The number of examples required to train a generative model highly depends on the unknown dimension of the data to generate. It should at the very least be larger than this unknown dimension. Since this dimension will highly vary depending on the population(s) and type of data (e.g. highly differentiated unlinked SNPs, fine scale local variation, ...) it would require training under multiple scenarios (different population, varying chunk size, varying sample size). A very large number of experiments (multiple training under the same scenario) would be needed to provide an answer and each experiment is computationally intense. Furthermore, this result would not be applicable to a new dataset. For direct application we thus suggest that it is more valuable to investigate the performances directly on the dataset of interest.

4)      Line 324—“real-alike” should be changed to “realistic”

We updated the word.
