# Supplementary material for: Creating artificial human genomes using generative neural networks
Source: PLoS Genet. 2021 Feb 4;17(2):e1009303. doi: 10.1371/journal.pgen.1009303 (PMC7861435; doi:10.1371/journal.pgen.1009303)
Supplement: Attachment — Submitted filename: response_to_reviewers_yelmen2020.docx [file pgen.1009303.s027.docx]

**Comments to the Authors:**

**Reviewer #1**: I reviewed a previous submission of this manuscript, and the authors have addressed essentially all of my prior comments. In particular, the exposition and the discussion section are greatly improved.

We thank the reviewer for highlighting the quality of our manuscript.

I have a few minor comments that I list below.

-- In discussion of using the coalescent as a generative model it may be helpful to discuss that the GAN is essentially learning something akin to the posterior predictive distribution: P(X | X_1, ..., X_N, \Theta) for observed haplotypes X_1,...,X_N and demographic parameters \Theta. Such conditional probabilities are notoriously hard to compute or even sample from under the coalescent, spawning a number of approximations (e.g., Li and Stephens' copying model; and Josh Paul's series of papers on Conditional Sampling Distributions), further highlighting the utility of the present method. The usual coalescent simulations, by contrast, are merely simulating under the prior, P(X | \Theta). Adding something to this effect in the discussion may help clarify some of the novelty and benefit of the present method, and clear up any confusion on why additional haplotypes cannot easily be simulated from the coalescent even if the demographic model is known.

We thank the reviewer for his comments. We added a sentence in the discussion explaining this difference. [To summarize, while the classical coalescent simulator...]

-- Mode collapse during GAN training has--to some extent--been solved by adding an entropic penalty (Prescribed Generative Adversarial Networks by Dieng, Ruiz, Blei, and Titsias). It is certainly not necessary to incorporate this into the present manuscript, but may be of future use to the authors.

We thank the reviewer for the suggestion and will investigate this in our future work. We also added this approach as a possible future task in the manuscript. [For some applications that depend on rare alleles, GAN models less sensitive to mode dropping may be a promising alternative (45,46,**47**).]

-- Lines 325-328 are unclear and difficult to understand.

Related to the other reviewer suggestions for removing the phenotype-genotype analysis, we excluded this part from the manuscript.

-- Line 378: The "this" in "we initially speculated that this" is unclear. I believe it's related to the decrease in performance for higher frequency alleles, but it currently reads like it's referring to the fact that increasing the number of AGs did not affect the results.

We updated the sentence to “We initially speculated that this **decrease** might be related to the disturbance in haplotypic structure…”

**Reviewer #2**: The authors have done a nice enough job on the revision, and I'm happy with most of it however I have a few sticking points still.

1) the comparison between simulated CEU and Estonians seems undesirable-- the authors are using 1000 Genomes data -- why not do a direct comparison to the CEU genomes in that dataset?

We thank the reviewer for their comments. In our work we wanted to compare  biological properties (LD, SFS, ability to blend within real data through PCA) of genomes obtained through coalescent simulations and GAN/RBM. To do this extent, unfortunately, we couldn’t rely on a single population (e.g. either just CEU or Estonians) because i) the full demographic models of Estonians is not yet clarified and ii) the CEU genomes available from the 1000 Genome Project are not sufficient to achieve a satisfactory learning within the 20,000 epochs we have decided to deploy for this study. Therefore we had to provide an imperfect comparison, demonstrating in practice the options available to a researcher interested in increasing the sample size of a population for which many genomes are available but the demographic model is unknown: either learn with GAN/RBM the information available from the empirical sample, or resort to a proxy population (CEU), for which demography is known but whose affinity to the study population (Estonians) is not perfect. We chose not to compare simulated CEU with real CEU from the 1000 Genomes to avoid replicating quality checks already reported in Gutenkunst et al. (Figure 3) and we also could not compare real CEU with the ones generated for the entire 1000 Genomes Project with GAN/RBM because there is not yet a robust way to select specific populations out of the AG dataset. In other words, we do not know explicitly which 1000 Genome AGs are CEU AGs, and this particular task will be part of future research. Finally, we would like to stress out again that simulating individuals with the coalescent model and the CEU parameters will not allow a one-to-one correspondence between the simulated and real CEU and will not allow merging the simulated dataset with a real dataset for downstream analyses, which is the core interest of our study.

Gutenkunst RN, Hernandez RD, Williamson SH, Bustamante CD. Inferring the joint demographic history of multiple populations from multidimensional SNP frequency data. PLoS Genet. 2009

2) again i find the phenotypic analysis to be too cursory for publication.

We removed the phenotypic analysis from the results and just kept a few sentences in the manuscript referring to supplementary material. [In addition, we showed that it might be possible to generate AGs with simple phenotypic traits through genotype data..]

**Reviewer #3**: This is my review of the revised submission of "Creating Artificial Human Genomes Using Generative Neural Networks". My primary concern with the initial submission was the insufficient discussion of the practical utility of the authors' approach to generating artificial genomes. This has been largely addressed in the revision but I do have several outstanding requests:

1. The authors have done little to clarify the utility of recapitulating in AGs the phenotype-genotype associations from real data. What could these be used for? Sharing more thoughts on this could help readers see the value of the authors' approach.

We thank the reviewer for their comments. We removed the phenotypic analysis from the results and just kept a few sentences in the manuscript referring to supplementary material. [In addition, we showed that it might be possible to generate AGs with simple phenotypic traits through genotype data..]

2. Please elaborate on the method's ability to create larger samples. The authors response on this issue seems to imply that multiple independent runs can be combined to create a sample with a larger number of genomes. In this case, our hope would be that the resulting artificial population sample would to some degree makes sense from an evolutionary standpoint, i.e. the genomes in that sample all implicitly belong to an ancestral recombination graph that is more or less drawn from the same distribution as that from which the GAN was trained. But if we take two AG samples and combine them to make a larger sample, will this be the case, or will they look like two different populations (or in the case of gene-scale AGs, two different loci) that were stitched together? I may be misunderstanding, but I do not think that the authors' response and revision addresses this at all. One way in which this could be tested is by making sure that FST between the two AG samples that were combined together is not elevated.

In its current form, we are still exploring what would be the best approach for creating whole genomes and our future studies will address this. Note that creating larger samples, i.e. with more individuals, is already possible and trivial with trained RBMs and GANs [...After the training is complete, it is possible to generate as many AGs as desired.]. Regarding the genome length, the simplest idea would be, through independent iterations, to generate multiple chunks (one for each “locus”) belonging to a **single uniform population** such as Estonians and stitch them together. If there is population structure in the training dataset, such as whole 1000 Genomes data, then it would be more challenging to combine the trained chunks and in that case, (a) conditioning generative models on group labels (as we had suggested [...conditional GAN/RBM methods to allow fine control over the composition of artificial datasets based on ... additional labels such as population names…] or (b) relying on FST as the reviewer suggests, are two options that should be investigated in future work. It is also important to note that for analyses solely based on overall allele frequency, it is not even required to create stitched genomes. It is possible to just generate multiple chunks for different regions of the genome and use them as they are. We added a section in the discussion related to these points. [It should be possible to create whole genomes by independently training and generating multiple chunks from different genomic regions using a single uniform population such as Estonians and stitching them together to create a longer, genome-like, sequence for each AG individual…]

3. I reiterate my request to include the Supplementary Text on RBMs in the main text. The authors' response about size limitations is not valid. Per the PLoS Gen website: "Manuscripts can be any length. There are no restrictions on word count, number of figures, or amount of supporting information."

There was indeed a confusion on our side regarding the restrictions and we have now added the RBM text to the manuscript.

4. Regarding the legend for Supplementary Figure 7: please more explicitly state that this is a scatter plot comparing real and artificial allele frequencies between ANALOGOUS polymorphisms in the two datasets.

We updated the legend to “Allele frequency comparison of **corresponding SNPs**…”

5. The authors' new results examining the RBM scheme that has reduced privacy loss are interesting. They should be included in the paper rather than simply stating that this RBM "had only a slight decrease in quality of the summary statistics" while sharing no data.

We added summary statistics to that part. [The correlation between generated and true allele frequencies was 0.92…]

6. The precise stopping criteria for GAN training were not added to the text as requested in my original review. Please rectify this.

As explained in Materials & Methods section, the main criterion was the visually coherent PCA results of AG and real genome combined datasets (see Figure 1 for an example). We additionally tested the possibility of using AATS score as a stopping criterion but the gain from additional training up to 30K epochs for AATS convergence was minimal as described in the manuscript. As visually coherent PCA results were achieved at 20K epochs for all datasets (i.e. generated and real individuals are projected in the same area of the space defined by the two first PCs), we opted to use AGs at this stage. However, it is possible to use AGs before or after 20K epoch point and future studies might explore other stopping criteria. We updated the section to clarify these better [Therefore, this study presents results for AGs generated at 20k epochs, since...].
